# Preclinical trials in Alzheimer’s disease: Sample size and effect size for behavioural and neuropathological outcomes in 5xFAD mice

**DOI:** 10.1371/journal.pone.0281003

**Published:** 2023-04-10

**Authors:** Mahvish Faisal, Jana Aid, Bekzod Nodirov, Benjamin Lee, Miriam A. Hickey

**Affiliations:** Department of Pharmacology, Institute of Biomedicine and Translational Medicine, University of Tartu, Tartu, Estonia

## Abstract

5xFAD transgenic (TG) mice are used widely in AD preclinical trials; however, data on sample sizes are largely unaddressed. We therefore performed estimates of sample sizes and effect sizes for typical behavioural and neuropathological outcome measures in TG 5xFAD mice, based upon data from single-sex (female) groups. Group-size estimates to detect normalisation of TG body weight to WT littermate levels at 5.5m of age were N = 9–15 depending upon algorithm. However, by 1 year of age, group sizes were small (N = 1 –<6), likely reflecting the large difference between genotypes at this age. To detect normalisation of TG open-field hyperactivity to WT levels at 13-14m, group sizes were also small (N = 6–8). Cued learning in the Morris water maze (MWM) was normal in Young TG mice (5m of age). Mild deficits were noted during MWM spatial learning and memory. MWM reversal learning and memory revealed greater impairment, and groups of up to 22 TG mice were estimated to detect normalisation to WT performance. In contrast, Aged TG mice (tested between 13 and 14m) failed to complete the visual learning (non-spatial) phase of MWM learning, likely due to a failure to recognise the platform as an escape. Estimates of group size to detect normalisation of this severe impairment were small (N = 6–9, depending upon algorithm). Other cognitive tests including spontaneous and forced alternation and novel-object recognition either failed to reveal deficits in TG mice or deficits were negligible. For neuropathological outcomes, plaque load, astrocytosis and microgliosis in frontal cortex and hippocampus were quantified in TG mice aged 2m, 4m and 6m. Sample-size estimates were ≤9 to detect the equivalent of a reduction in plaque load to the level of 2m-old TG mice or the equivalent of normalisation of neuroinflammation outcomes. However, for a smaller effect size of 30%, larger groups of up to 21 mice were estimated. In light of published guidelines on preclinical trial design, these data may be used to provide provisional sample sizes and optimise preclinical trials in 5xFAD TG mice.

## Introduction

By 2050, cases of dementia are expected to almost triple from 2019 levels [1, 2]. Alzheimer’s disease (AD) is the leading cause of dementia [3] and the highest-ranking cause of disability-adjusted life years of the neurodegenerative diseases [4]. Although many possible treatments for AD are in development [5, 6], drug approval rates are typically low for the nervous system [7] and a recent approval of Aducanumab [8] has proven controversial [9, 10].

Preclinical trials in animal models are critical for the development of new drugs and also validation of disease mechanisms. Recent editorials and reviews have highlighted several important issues to address in rodent preclinical trials including trial design, trial registration and transparent reporting [11–13]. Specific areas to address include e.g., blinding, randomisation, inclusion/exclusion criteria, prior design of statistical analyses and sample size, and resources such as the Experimental Design Assistant have been developed to assist [14, 15].

Sample-size estimations are required for ethical approval and enable appropriate statistical power to detect an expected treatment effect. However, many preclinical therapeutic trials in AD transgenic mice show no sample-size calculation [16]. The authors suggested this may underlie the typically small sample sizes used (N<10) [16], and indeed, „small-study effects”have been noted in AD preclinical research [17]. Similar issues with respect to group sizes and sample-size calculations have been noted in preclinical trials in rodent models of Parkinson’s disease [18] and in rodent fear-conditioning [19]. Underpowered preclinical studies were identified as the single most important factor that contributed to failure of a therapeutic in a clinical trial in human ALS patients [20], and high-impact guidelines, including ARRIVE guidelines, recommend that sample-size estimates be conducted for preclinical testing [21, 22]. Nevertheless, an expected effect size is difficult to predict and the (clinical) significance of a particular effect size is difficult to estimate [23, 24]. Here, we have focused chiefly upon normalisation to wildtype levels, the assumed largest expected effect size for a potential therapeutic, and also a smaller effect size of 30% improvement in TG outcomes where possible. This smaller effect size may be relevant for a test agent of unknown efficacy [25]. We provide effect sizes for each outcome, together with its power, and then use several different, freely available resources for our sample size estimates.

5xFAD mice are a very well-known mouse model of Alzheimer’s disease [26]. They have been used by many researchers worldwide to study basic mechanisms underlying AD pathophysiology and also in preclinical trials. Our aim was to estimate sample sizes required to detect different effect sizes using outcomes from behavioural and neuropathological assays at ages where amyloid load is well-characterised [26]. The behavioural and neuropathological assays used are well-known and characterised for mice (automated open field [27, 28]; spontaneous alternation [29]; forced alternation [30, 31], novel object recognition [32]; Morris water maze [33]; neuropathology [26, 34–36]) but their relative ability to detect cognitive deficits in 5xFAD mice are inconsistent in the field. Indeed, recent data suggests that the most robust behavioural finding in 5xFAD mice is an increase in activity, rather than a change in cognition [37].

Assessments of relative efficacy of well-used tests are becoming more common in AD preclinical research [37–39] but sample sizes remain unclear. The experiments outlined here use standard protocols to provide provisional sample sizes to detect treatment effects in 5xFAD mice, one of the most widely used mouse models of AD.

## Materials and methods

### Mouse husbandry

Male transgenic (TG) 5xFAD mice (034840-JAX; B6SJL-Tg(APPSwFlLon,PSEN1*M146L*L286V)6799Vas/Mmjax) and female B6SJLF1 mice were purchased from Jackson laboratories (Bar Harbor, ME). Mice were group-housed with access to ad lib food (V1534-300, ssniff Spezialdiäten GmbH) and water (reverse osmosis-treated, and UV sterilised) and lights were set to 7am on 7pm off. Pups were weaned at approximately 3 weeks of age and group-housed in same-sex, mixed-genotype, mixed-litter cages of 8–10 animals with one cage being n = 4 (n = 2 TG, N = 2 WT). Our paper adheres with ARRIVE guidelines [40]. Authorisation to perform experiments was provided by the Estonian Animal Welfare authorisation committee, licence numbers 175 and 189, according to the EU Directive 2010/63/EU.

### Genotyping

Mice were genotyped for the PSEN transgene, wildtype (WT) APP gene and also the phosphodiesterase-6b retinal degeneration-1 (Pde6brd1) allele by PCR (see Table 1 for primers, obtained from Tag Copenhagen, Frederiksberg, DN) using tail samples obtained from neonates between the ages of P3 and P10. TG/mutant and wildtype controls and a water control were run in every PCR. DreamTaq PCR Master Mix (2X) (ThermoFisher Scientific) was used for PCR. Mice that were homozygous recessive for Pde6brd1 were not used. Cycling conditions for AD status: 95°C for 5 mins followed by 40 cycles of 94°C, 48°C, 72°C for 45s, 30s, 90s, respectively, followed by a final extension at 72°C for 10minutes. Cycling conditions for Pde6brd1 status: 94°C for 2mins followed by 28 cycles of 94°C for 15s, 57°C for 15s and 72°C for 10s with a final extension time of 72°C for 2 mins.

**Table 1:** Primer sequences.

Name	Sequence	Size (bp) [citation(s)]
**PSN1 forward**	AAT AGA GAA CGG CAG GAG CA	608 [41–43]
**PSN2 reverse**	GCC ATG AGG GCA CTA ATC AT	
**WT APP forward**	CTA GGC CAC AGA ATT GAA AGA TCT	324 [42]
**WT APP reverse**	GTA GGT GGA AAT TCT AGC ATC ATC C	
**RD1 (mutant)**	AAG CTA GCT GCA GTA ACG CCA TTT	560 [42]
**RD2 (WT)**	ACC TGC ATG TGA ACC CAG TAT TCT ATC	240 [42]
**RD3 (common)**	CTA CAG CCC CTC TCC AAG GTT TAT AG	

### Cohorts tested

All behavioural analyses were conducted during the light phase of the light cycle (between 9am and 2pm). Male mice were not used because male transgenics exhibit a delayed disease progression compared with female transgenic mice [26]. Mice exhibiting stereotypy (continuously doing backflips or continuous jumping in the cage) or less than 16g were excluded from testing (N = 2 TG). Mice were handled to reduce anxiety and were weighed regularly. Behavioural testing was conducted blinded to genotype. For all behavioural testing, mice were acclimatised to the testing room for 20-30mins. For video-based automated analysis of behaviour, white, beige and grey fur was coloured using human hair spray (dark brown or black, L’Oréal).

#### Young cohort

Female transgenic and wild-type mice were tested, together (Young cohort: N = 14 WT, N = 16 TG). Mice were tested for spontaneous activity in an open field (136±2d mean ± sem; all mice on sasssme day), spontaneous alternation in the spontaneous alternation (age 137±2d; all mice on same day), novel object recognition memory (140±3d; mice divided over two days), spatial memory in the Morris water maze (all mice tested, together, over a 3-week period, see protocol below, beginning at 148±2d and ending at 169±2d) and forced alternation (189±3d; testing divided over three days).

#### Aged cohort

Female mice were tested, together (Aged cohort: WT N = 17, TG N = 12). Mice were tested for spontaneous activity in an open field (406±4d mean ± sem; all mice on same day), for spatial memory in the Morris water maze (all mice tested, together, over a 4-day period, see protocol below, beginning at 435±4d and ending at 438±4d) and for spontaneous alternation (442±4d; all mice on same day).

### Behavioural testing

#### Open field

Spontaneous activity in a novel environment was analysed using Noldus Phenotyper cages equipped with Ethovision XT V11 (Noldus, Wageningen, Netherlands). Mice were placed into the Phenotyper cages singly and their activity recorded for 1 hour. Outcome measures, generated automatically by the software, included distance travelled and speed. For N = 2 mice per genotype in the Young cohort, no data was collected for more than 30% of timebins and they were not included in analyses.

#### Spontaneous alternation

Mice were placed into the centre of the maze (3 arms at a 120° angle from each other, arms 30cm long and 10cm wide; walls and floor opaque and 10cm high) and their behaviour video-recorded for 5 min [39] or 8 minutes [26, 44, 45] for subsequent analysis. Visual cues in the form of room furniture, constant position of the experimenters etc., were available to the mice. Analysis of videos was conducted blinded. An entry was defined as when the hindquarters entered an arm. The number of entries, the arm entered, triplets (e.g., ABC, CBA, ACB) and working memory (re-entries within a triplet, i.e., unsuccessful triplet) errors were quantified from videos by a blinded observer. The apparatus was cleaned with 70% ethanol and dried thoroughly, between tests. N = 1 TG mice from the Young cohort and N = 2 TG mice from the Aged cohort did not reach entry number threshold of 10 and were not included in analyses.

#### Forced alternation

A T-maze, constructed from black Perspex walls and a white floor was used (stem: 30cm long, 10cm wide, 20cm high; distal arms each 30cm long, 10cm wide and 20cm high; Pleksiklaas OÜ, Tartu, EE). Animals were placed into the start (home, stem of the T) arm and allowed to explore the apparatus freely over a period of 5 minutes. One of the distal arms was blocked off during this phase (pseudorandomly assigned per mouse using Excel rand function). After 1 hour, the mouse was placed back into the start arm, and again allowed to explore freely over a period of 5 minutes (all arms now available for exploration). Visual cues in the form of room furniture, constant position of the experimenters etc., were available to the mice. Lighting was set to 20–40 lux at the centre of the maze. The apparatus was cleaned with 70% ethanol and dried thoroughly, between tests. The total distance travelled (using ezTrack [46]), preference index (entries into novel arm / total entries) and difference index (entries into novel arm minus entries into familiar arm) was quantified and compared. A threshold of 10 entries or more activity was required for use of behavioural data for analysis. Only 7 WT mice (out of 14) and 5 TG mice (out of 16) achieved this threshold.

#### Novel-object recognition

Testing was as per [32, 47]. The testing arena was a box with black walls and white floor made of Perspex (25cm x 25cm x 30 cm high: Pleksiklaas OÜ, Tartu, EE). Mice were placed into the box on day 1, with no objects, for habituation (habituation; 5 minutes). Behaviour was video recorded for analysis. On day 2, familiarisation and testing took place. During familiarisation, a pair of identical objects were pseudorandomly assigned to individual mice (using Excel rand function), which were placed into the box near the northwest and northeast corners. The mouse was then placed into the south end of the box facing away from the objects and behaviour recorded over a period of 5 minutes [32, 47]. Two sets of objects were used for novel object recognition: one set of identical objects were small, round, lidded jars filled with sand with duct tape around them to provide texture; the second set of identical objects were small, green, glass, hexagonal candle holders. No difference in baseline exploration of objects was found, no intrinsic preference for either set of objects was found and no genotype-dependent exploration of either set of objects found. Three hours later, during the testing phase, the final identical member of the object triplet (familiar object) was placed into one of the corners (randomly assigned) and one novel object (a jar if habituated to the candle holder; a candle holder if habituated to the jar) placed in the other corner. The mouse was then placed into the box at the south end, facing away from the objects and behaviour recorded over a period of 5 minutes. Light was approximately 20 lux at the centre of the box. The apparatus and objects were cleaned with 70% ethanol and dried thoroughly, between tests. Behaviour was analysed from videos by a blinded observer. Exploration was defined as when the mouse sniffed the object or touched it while looking at it at a distance of 2cm or less between mouse and object. Climbing was not considered exploration [47]. A threshold of 20s exploration during familiarisation was required for mouse to be included in data analysis [47]; N = 2 WT and N = 1 TG did not reach threshold. Outcome measures included preference score (time spent exploring novel object/total time spent exploring), difference score (time spent exploring novel object minus time spent exploring familiar object) and discrimination index (difference score/total time spent exploring objects) [32]. This test was not examined in the Aged cohort.

#### Morris water maze: Young cohort

The water maze (diameter 140 cm, height 45 cm) was filled with water (22°C), that was then coloured using tempura white paint to obscure the platform position. Visual cues were placed surrounding the maze. The trial length for all trials was 60s. If an animal failed to find the platform within 60s, they were guided gently to it. For trials where a platform was present, the platform was placed at 1cm below the surface of the water and threshold for successful location of the platform was 5.2s on the platform. Mice were trained to stay on the platform for 10-15s before being removed and placed into a warmed cage. Inter-trial intervals were approximately 10 minutes. The order of testing was the same for individual mice within a cage, but order of cages was changed daily.

For cued learning, mice were trained (4 trials per day over a period of 5d) to locate a submerged platform marked with a highly salient visual cue. Starting positions and platform positions varied with trial, according to [33]. Despite extensive guidance, N = 1 WT mouse failed to demonstrate an ability to find the cued platform; data from this mouse was not included in the analyses.

Spatial learning began on the 8^th^ day. For spatial learning, mice were trained over 4 trials per day, for 6 days, to find a submerged platform that remained fixed in the southwest position. Starting positions varied with trial according to [33] (with the addition of D6, starting positions trial 1: SE, trial 2:NW, trial 3: E, trial 4:N). On the 7th day of spatial learning, the platform was removed, and mice placed in the pool at a novel start-site (NE) for probe testing.

Reversal learning began on the 15^th^ day. The mice were given 4 trials per day over a period of 6 days to learn a new fixed platform position (NE). Starting positions varied with trial according to [33] (with the addition of D6, starting positions trial 1: NW, trial 2: W, trial 3: SE, trial 4: S). On the 7th day of reversal learning, the platform was removed, and mice placed in the pool at a novel start-site (SW) for probe testing.

#### Morris water maze: Aged cohort

Protocols were as per guidelines provided in Vorhees and Williams [33] and as described above. However, we had to make several adjustments to assist the frail TG mice, including 1) raising the water temperature: 23–24°C, 2) lowering the platform position to 2cm below the water surface to enable the TG mice to climb on, 3) increasing the inter-trial interval to approximately 60 minutes, 4) lowering the number of trials per day–on day 1, the number of trials was 4 but for subsequent days 2–4, we used 3 trials per day. Finally, mice were tested over 4 days of cued learning (visual learning) only. Spatial learning and reversal learning phases were not conducted as transgenic mice did not achieve sufficient success during cued learning. N = 1 WT showed thigmotaxis (swam following the wall and consistently less than 5cm from wall [33]) and was removed from the analysis.

#### Morris water maze: Analysis

For analysis, Ethovision XT V8 (Noldus, Wageningen, Netherlands) was used to calculate latencies to find platform, quadrant occupancies, Gallagher’s proximity, velocity, distance, time on platform and frequency of platform crossings.

### Pathological analysis

A series of mice at 2, 4 and 6 months of age were analysed (N = 3–4 [42, 48, 49] female wildtype and transgenic littermates per age). Mice were euthanised by cervical dislocation and decapitation. Brains were dissected out and divided into hemispheres and one hemisphere was placed in fresh 4% paraformaldehyde for post-fixing. Samples for post-fixing were incubated at 4°C, with rocking, for 48-72hrs. Samples were then placed in 30% sucrose for a further 48-72hrs and then briefly washed with 0.01M PBS, excess liquid dried off and then they were snap frozen in liquid nitrogen. Samples were stored at minus 80°C. Serial sagittal cryosections (40μm) were taken and placed in cryoprotectant and stored at minus 20°C until processing. No plaques are observed in non-TG mice from this line of mice [26] and so WT mice were included in astrocyte and microglial analyses only.

#### Congo red staining

Three sections per mouse, approximately -2.0mm lateral from bregma, were stained for Congo red as previously described [35]. Briefly, sections were washed in 0.01M TB for 3x5 minutes and then mounted onto gelatin-coated glass slides and dried overnight. On the following day, sections were washed in dH_2_0 (30s) then placed in saturated NaCl (NaCl is added to 80% EtOH while stirring until a layer of approximately 5mm is obtained) for 20 minutes. Slides were then placed in Congo red solution for 30 minutes (0.2% Congo red in saturated NaCl, filtered prior to use). Slides were then brought through dehydration steps (8 dips in 95% ethanol, 3 x 5 minutes in xylene) and coverslipped. Photomicrographs were taken at x20 using cellSens Entry, V2.2 software (Olympus Life Science, Center Valley, Pennsylvania) on an Olympus IX70 microscope. Images quantified by a blinded observer for size and number of plaques per field of view at the hippocampus (DG, CA1, CA2/3, subiculum) and frontal cortex. Briefly, the number of plaques was quantified using cell counter in ImageJ (FIJI), and for plaque size, a grid was placed on the image and the area of any plaque contacting lines on the grid (1500μm^2^, random offset) was quantified in ImageJ (FIJI) to a maximum number of 10 per image (1 image per region of interest per section).

#### Fluorojade C staining

For Fluorojade C (FJC) staining [36], two sections per mouse, approximately -2.0mm lateral from bregma, were mounted onto gelatin-coated slides, dried overnight and then washed in 0.01M PBS for 1 min then incubated in KMnO_4_ (0.06% in 0.01M PBS) for 20mins and incubated in FJC for 20 minutes (0.0001% in 0.01M PBS + 0.1% acetic acid). Sections were then washed in 0.01M PBS for 3x1min, dehydrated and defatted for coverslipping. Photomicrographs (1 image per region of interest per section) were taken at x10 using Zen software on an LSM 780 confocal microscope (ex 488nm, em 505-550nm). Images were batch-processed in ImageJ (FIJI) using the Intermodes thresholding algorithm, followed by despeckling and then analysis of particles greater than 5μm^2^.

#### Immunocytochemistry

Free-floating sections were processed according to standard protocols. Two lateral (2.6mm lateral of Bregma) and two medial (1.5mm lateral of Bregma) sections were used per mouse.

For IBA1, sections were washed (3 x 5mins 0.01M PBS) and then endogenous peroxidases inactivated (1% H_2_O_2_ in 0.5% Triton X-100 in PBS; 20 min). Sections were then blocked (5% donkey serum (Jackson laboratories) in 0.5% TX-100 in 0.01M PBS; 30 minutes) and incubated in primary antibody overnight (Abcam Iba1 ab5076; 1:1000 in blocking solution). On the following day, sections were washed (3 x 5mins 0.01M PBS) and incubated in secondary antibody (donkey anti-goat 705-065-147 Jackson ImmunoResearch; 1:200 in blocking solution) for 2 hours. Following washing (3 x 5mins 0.01M PBS), sections were incubated in Vectastain Elite ABC Reagent in PBS containing 0.2% Triton X-100 for 2 h, according to manufacturer instructions. Sections were washed (3 x 5mins 0.01M PBS) and then developed in 0.03% 3-3-diaminobenzidine tetrahydrochloride containing 0.0006% H_2_O_2_ in 0.05 M Tris buffer, pH 7.6. Development was monitored carefully and then sections washed in 0.01M TB for 4 x 5minutes. Sections were then mounted onto gelatin-coated slides, dehydrated and defatted and then coverslipped for photography. Control sections were run in parallel that were not exposed to primary antibody: no staining was observed in these sections.

For GFAP, sections were washed (3 x 5mins 0.01M PBS) and then blocked (5% goat serum (Jackson laboratories) in 0.5% TX-100 in 0.01M PBS; 30 minutes) and incubated in primary antibody overnight (GFAP, Sigma Aldrich FLJ45472; 1:500 in blocking solution). On the following day, sections were washed (3 x 5mins 0.01M PBS) and incubated in secondary antibody (goat anti-rabbit 111-585-003 Jackson ImmunoResearch; 1:500 in blocking solution) for 2 hours. Following washing (3 x 5mins 0.01M PBS), sections were incubated in Hoechst (1μg/ml) for 10 minutes, washed 1 x 5mins in 0.01M TB and then mounted onto gelatin-coated slides and coverslipped using aqueous mounting medium. Control sections that were run in parallel that were not exposed to primary antibody showed no staining.

#### Image analysis for immunohistochemistry

*IBA1*. Photomicrographs of subiculum and frontal cortex layers V-VIa were taken at x20 using cellSens Entry, V2.2 software (Olympus Life Science, Center Valley, Pennsylvania) on an Olympus IX70 microscope. To ensure consistency, all pictures were taken using the same settings, having calibrated brightness across the field of view and white-balanced the camera. For analysis, images were processed as previously published with small modifications [34]. Briefly, using ImageJ batch processing, images were FFT bandpass filtered, converted to grayscale, brightness and contrast were adjusted automatically, then an unsharp mask was run twice, images were then despeckled, and converted to binary using the RenyiEntry for automated thresholding. The final images were then despeckled, and the close and the remove outliers plugins were used to close objects and smooth final objects. All particles greater than 30μm^2^ and away from edges were measured, and mean particle size per mouse used to generate group means.

*GFAP*. Z-stack photomicrographs through the depth of the subiculum and of frontal somatosensory cortex layers V and VIa using an LSM780 confocal (x20; 0.92 x 0.92 x 2.04μm per pixel; frame size: 472.33 x 472.33 μm;). Images were batch-processed in ImageJ. Briefly, colours were split and the GFAP channel made into maximum-intensity projections, converted to 8-bit, auto-thresholded (Maximum Entropy algorithm) and percent area per image quantified to generate means per mouse and then group means.

### Statistical analyses

All analyses were conducted blinded to genotype and then the code broken for generating graphs and for statistics. Individual data points as well as mean values +/- standard error of the mean are shown where possible. Critical values were set to 0.05. Body weights from Young and Aged cohorts were combined for analysis and analysed using a mixed-effects ANOVA with Geisser-Greenhouse’s epsilon followed by Šídák’s multiple comparisons tests. To compare one factor between two separate groups, unpaired T-tests were used (e.g., open field total distance moved over 1 hour, e.g., open field velocity over 1 hour). Where one factor was compared over time within a particular group, 1-way ANOVAs with repeated measures were used followed by appropriate post-hoc testing. Two-way ANOVAs with Geisser-Greenhouse’s epsilon were used to compare data where there were two factors (e.g., genotype and time) and were followed by appropriate post-hoc tests, e.g., Gallagher’s proximity over days of learning in the Morris water maze. In the case of missing data, mixed-effects ANOVAs were used instead. Post-hoc tests were Šídák’s multiple comparisons tests for between-group analyses and Tukey’s multiple comparisons tests for within-group analyses. Three-way ANOVAs with repeated measures and followed by Tukey’s multiple comparisons test were used for GFAP and IBA1 immunostaining data. For Novel object recognition, one-sample T-tests were used to compare performances to theoretical values. To compare the mean number of trials showing “bumps” per mouse per group on the final day of MWM testing in the Aged cohort, a Mann Whitney U test was used because data were non-parametric.

GraphPad Prism V9.3.1 was used for the statistical analyses for basic outcome measures; a mouse was considered to be the experimental unit. ClinCalc [50] was used for post-hoc power estimations, Cohen’s D was calculated as per [51]. Sample-size estimations were based upon two-tailed hypotheses, and power was set to 80% except where noted. Matlab [52], ClinCalc [53], BioMath [54] and G-Power [55] were used to determine sample sizes. Sample-size estimates assumed the same group size (1:1) and standard deviation. Power calculations, effect sizes and sample-size calculations are only calculated where robust between-genotype effects were observed. Data are available within S1 File.

## Results

### Body weight

**Fig 1:**
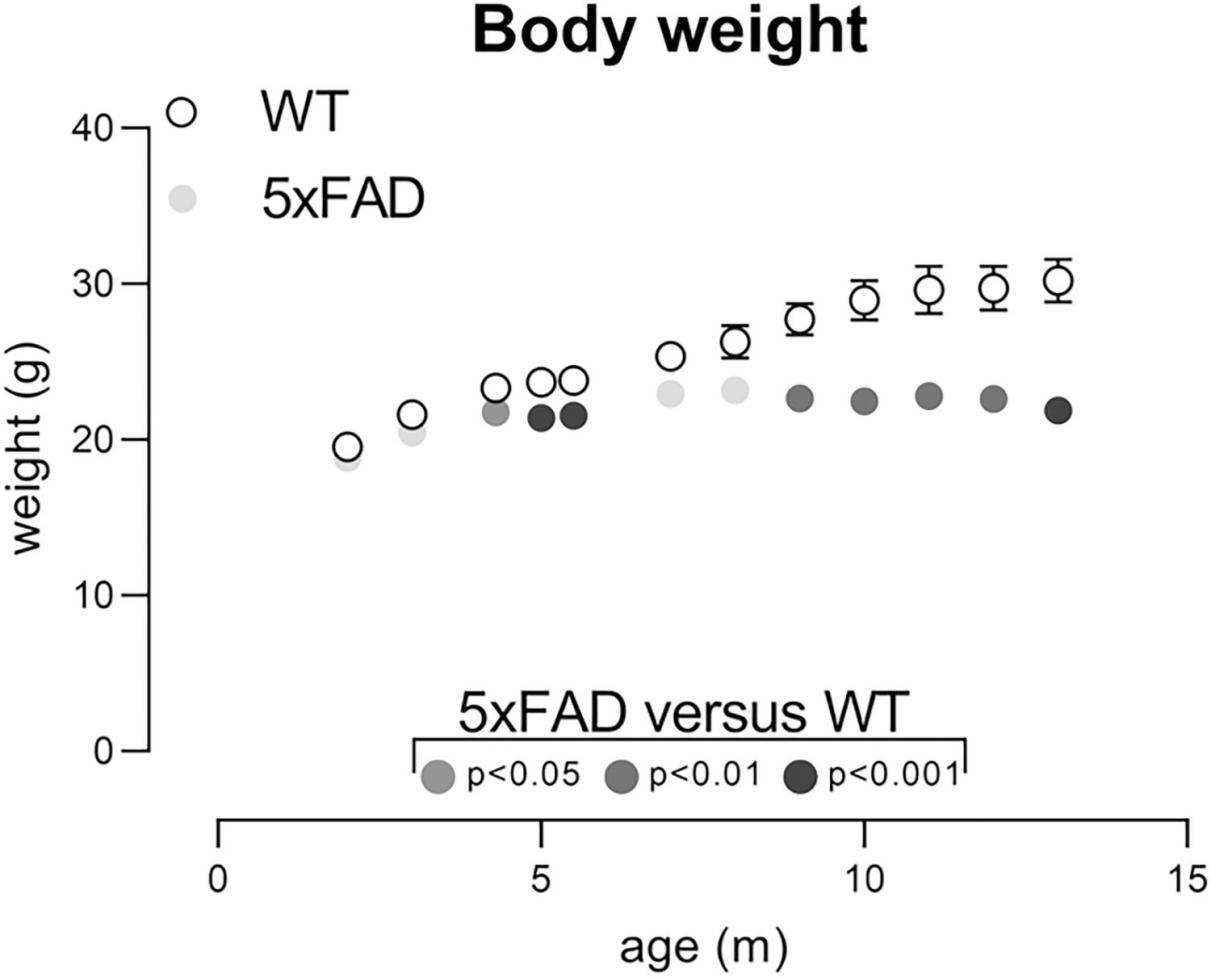
Body weights of WT and TG mice. Body weights from Young (N = 14 WT, N = 16 TG)) and Aged (N = 17 WT, N = 12 TG) cohorts were combined for analysis and analysed using mixed-effects ANOVA followed by Šídák’s multiple comparisons tests. WT mice gain weight throughout but 5xFAD TG mice reach maximum weight by 8m. The colours of the symbols used for TG mice show whether their weights are similar to their WT littermates (light grey) or are significantly different from their WT littermates with increasingly darker shades denoting increasingly significant differences. Data are of group mean ± sem. but if errors are less than the size of the symbol, they are not shown.

Reproducing previous data [37, 38, 56, 57], body weights in 5xFAD females failed to increase as much as WT littermates and began to differ significantly from WTs by approximately 4 months (Fig 1; body weights from Young and Aged cohort combined for analysis; age x genotype (F(11,416) = 14.9, p<0.0001). WT females continued to gain weight throughout with AUC analysis showing peak weight at 13m; approximately 30g. TG female peak weight occurred at 8m; approximately 23g. The effect size of weight differences between WT and TG mice was higher for 1-year-old mice (Table 2). At 5.5m, 9–15 mice (depending upon algorithm used to calculate sample size) would be required to detect a treatment effect of bringing weights back to WT levels (Table 3). The theoretical sample sizes required to detect a treatment effect of bringing weight back to WT levels are very small at 1 year of age (1 to <6), likely reflecting this larger difference between genotypes (Table 3). Thus, group sizes for this endpoint at 1 year of age are relatively small if the expected effect size for a particular agent is large (>30%).

**Table 2:** Effect size and post-hoc power of pathological, behavioural and general health outcomes.

Outcome measure	Cohen’s D [51]	Post-hoc power [50] (alpha = 0.05)
**Neuropathological outcome measures**		
Plaque load: Congo red-positive plaque density in frontal somatomotor cortex at 4m, to bring to level of 2m-old TG mice	4.0	99.9%
Plaque load: Congo red-positive plaque density in CA2/3 at 4m, to bring to level of 2m-old TG mice	-^a^	100%
Plaque load: Congo red-positive plaque density in dentate gyrus at 4m, to bring to level of 2m-old TG mice	-^a^	100%
Plaque load: Congo red-positive plaque density in subiculum at 4m, to bring to level of 2m-old TG mice	6	100%
^b^Microgliosis: IBA1-positive particle size in frontal cortex at 4m, to bring to level of 4m-old WT mice	4.2	99.9%
^b^Microgliosis: IBA1-positive particle size in frontal cortex at 6m, to bring to level of 6m-old WT mice	3.6	99.4%
Microgliosis: IBA1-positive particle size in subiculum at 4m, to bring to level of 4m-old WT mice	2.9	94.8%
Microgliosis: IBA1-positive particle size in subiculum at 6m, to bring to level of 6m-old WT mice	2.0	81.8%
Astrogliosis: GFAP-positive percent area in frontal cortex at 4m, to bring to level of 4m-old WT mice	4.1	99.9%
Astrogliosis: GFAP-positive percent area in frontal cortex at 6m, to bring to level of 6m-old WT mice	3.2	99.8%
Astrogliosis: GFAP-positive percent area in in subiculum at 4m, to bring to level of 4m-old WT mice	5.4	100%
Astrogliosis: GFAP-positive percent area in in subiculum at 6m, to bring to level of 6m-old WT mice	12	100%
**Behavioural or general health outcome measures**		
Body weight: Weight at 5.5m, to bring to level of 5.5m-old WT mice	1.1	99%
Body weight: Weight at 12m, to bring to level of 12m-old WT mice	1.6	100%
Open field: Distance travelled in Aged TG mice, to bring to level of WT mice	1.2	92%
MWM, Reversal Probe trial, proximity index, 1^st^ 10s: Young cohort, to bring to level of WT mice	1.1	88%
MWM, Reversal Probe trial, proximity index, entire trial: Young cohort, to bring to level of WT mice	1.1	88%
MWM, Reversal Probe trial, occupancy of NE quadrant, entire trial: Young cohort, to bring to level of WT mice	1.3	91%
MWM, Reversal Probe trial, occupancy of NE quadrant, 1st 10s: Young cohort, to bring to level of WT mice	1.2	92%
MWM, Cued learning proximity index: Aged cohort, to bring to level of WT mice	1.6	98.3%

^a^Not possible to calculate because one group has no standard deviation (no plaques were detected in CA2/3 or in DG at 2m in TG mice).

^b^Although this outcome measure did not survive post-hoc testing following our three-way ANOVA, it is provided for completion, as T-tests for this outcome may appropriate for some preclinical trials.

**Table 3:** Group sizes required to detect treatment effects for neuropathological, behavioural or general health outcome measures.

Outcome measure	Group size required to detect improvement to levels equivalent to WTs α = 0.05 80% power, except where noted Group sizes 1:1			
**Neuropathological outcome measures**				
	Matlab	ClinCalc	Biomath	G-Power
Plaque load: Congo red-positive plaque density in frontal somatomotor cortex at 4m, to bring to level of 2m-old TG mice	4	2	<6	4
Plaque load: Congo red-positive plaque density in CA2/3 at 4m, to bring to level of 2m-old TG mice	4	2	<6	3
Plaque load: Congo red-positive plaque density in dentate gyrus at 4m, to bring to level of 2m-old TG mice	3	1	<6	3
Plaque load: Congo red-positive plaque density in subiculum at 4m, to bring to level of 2m-old TG mice	3	1	<6	3
^a^Microgliosis: IBA1-positive particle size in frontal cortex at 4m, to bring to level of 4m-old WT mice	4	2	<6	3
^a^Microgliosis: IBA1-positive particle size in frontal cortex at 6m, to bring to level of 6m-old WT mice	4	2	<6	4
Microgliosis: IBA1-positive particle size in subiculum at 4m, to bring to level of 4m-old WT mice	5	4	<6	5
Microgliosis: IBA1-positive particle size in subiculum at 6m, to bring to level of 6m-old WT mice	6	7	9	9
Astrogliosis: GFAP-positive percent area in frontal cortex at 4m, to bring to level of 4m-old WT mice	4	2	<6	4
Astrogliosis: GFAP-positive percent area in frontal cortex at 6m, to bring to level of 6m-old WT mice	4	3	<6	4
Astrogliosis: GFAP-positive percent area in in subiculum at 4m, to bring to level of 4m-old WT mice	3	1(95% power)	<6	2
Astrogliosis: GFAP-positive percent area in in subiculum at 6m, to bring to level of 6m-old WT mice	3	0^b^	<6	2
**Behavioural or general health outcome measures**				
Body weight: Weight at 5.5m, to bring to level of 5.5m-old WT mice	9	14	15	12
Body weight: Weight at 12m, to bring to level of 12m-old WT mice	3	1	<6	3
Open field: Distance travelled in Aged TG mice, to bring to level of WT mice	6	6	8	8
MWM, Reversal Probe trial, proximity index, 1^st^ 10s: Young cohort, to bring to level of WT mice	13	21	22	22
MWM, Reversal Probe trial, proximity index, entire trial: Young cohort, to bring to level of WT mice	12	18	20	22
MWM, Reversal Probe trial, occupancy of NE quadrant, 1st 10s: Young cohort, to bring to level of WT mice	9	12	14	14
MWM, Reversal Probe trial, occupancy of NE quadrant, entire trial: Young cohort, to bring to level of WT mice	8	11	12	12
MWM, Cued learning proximity index on Day 4: Aged cohort, to bring to level of WT mice	6	7	9	9

Group sizes required to enable use of a T-test to detect a difference equivalent to that depicted in the left-most column: Mathworks: [52], ClinCalc [53], Biomath [54], G-Power [55]

^a^Although this outcome measure did not survive post-hoc testing following our three-way ANOVA, it is provided for completion, as T-tests for this outcome may appropriate for some preclinical trials.

^b^Likely a statistical anomaly due to large differences between groups.

### Behavioural endpoints: Activity

**Fig 2:**
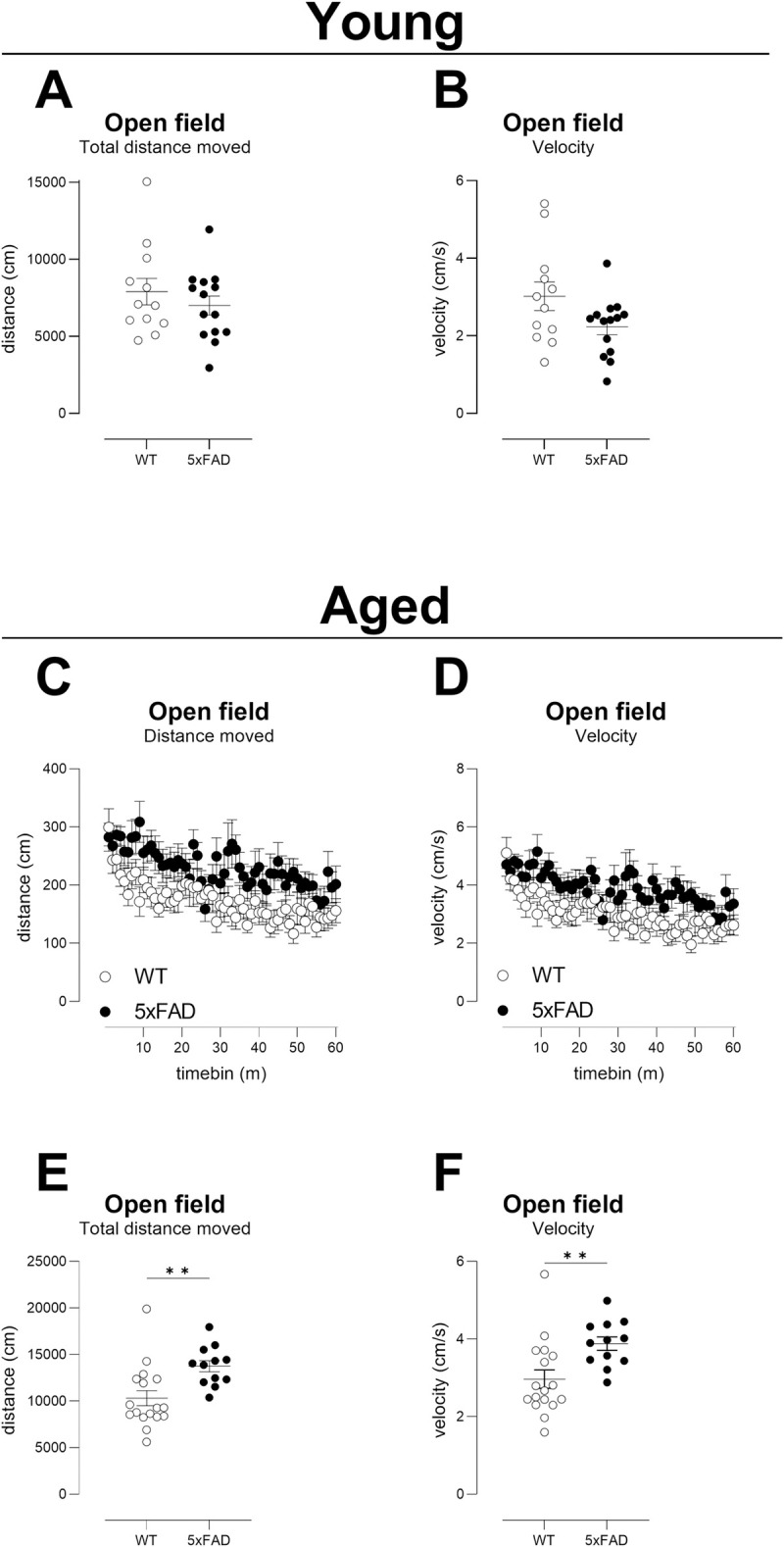
Spontaneous activity in a novel environment. Activity in a novel environment (open field activity) over a period of 1 hour. No difference was detected between genotypes in the Young cohort and we note a large within-group variability at this age (A, B). C-F: By 13m of age, 5xFAD TG mice are hyperactive. Graphs C and D show per-minute analyses (symbols are of group means±sem), but no individual timepoints were significantly different between genotypes. E, F: Analysis of total activity over the 1-hour period was a robust and more useful outcome measure for detecting treatment effects than per-minute analysis. A, B, E, F: Individual mice are shown as black filled circles (TG) or open circles (WT) with lines depicting mean ± sem. C, D: mean ± sem shown. Group sizes: Young: N = 12 WT, N = 14 TG; Aged: N = 17 WT, N = 12 TG.

The Young cohort showed no differences in horizontal activity (Fig 2A) or speed (Fig 2B). We noted a high within-group variability in these young mice (distance SD/mean: WT = 37%, TG = 33% versus 11–12%, respectively, at 1 year of age). Sample-size estimations were not calculated.

In the Aged cohort, TG mice showed increased horizontal activity (Fig 2C, effect of genotype F(1,27) = 9.6, p<0.01) and increased speed of movement (Fig 2D, effect of genotype F(1,27) = 7.8, p<0.01). However, post-hoc tests showed no significant differences between genotypes at any individual “per minute” timepoint (Fig 2C and 2D genotype x time interaction F (59, 1588) = 1.0, ns for distance and F (59, 1588) = 1.0, ns for speed), suggesting that analysis of short timepoints is not optimal for determining treatment effects. When analysing total activities using T-tests, differences were as robust as the genotype factor from the ANOVAs (Fig 2E and 2F; p<0.01 for each outcome measure WT versus TG). Post-hoc power, *per se*, can be problematic because, statistically, it remains possible that the null hypothesis is correct [58]. However, as our data reproduces recent findings from others [37], the null hypothesis of there being no difference between genotypes is less likely. Thus, post-hoc power for the difference in distance travelled is high (Table 2) and only 6–8 mice would be required to detect a treatment effect of bringing distance travelled to WT levels (Table 3).

### Behavioural endpoints: Cognitive

#### Spontaneous alternation

**Fig 3:**
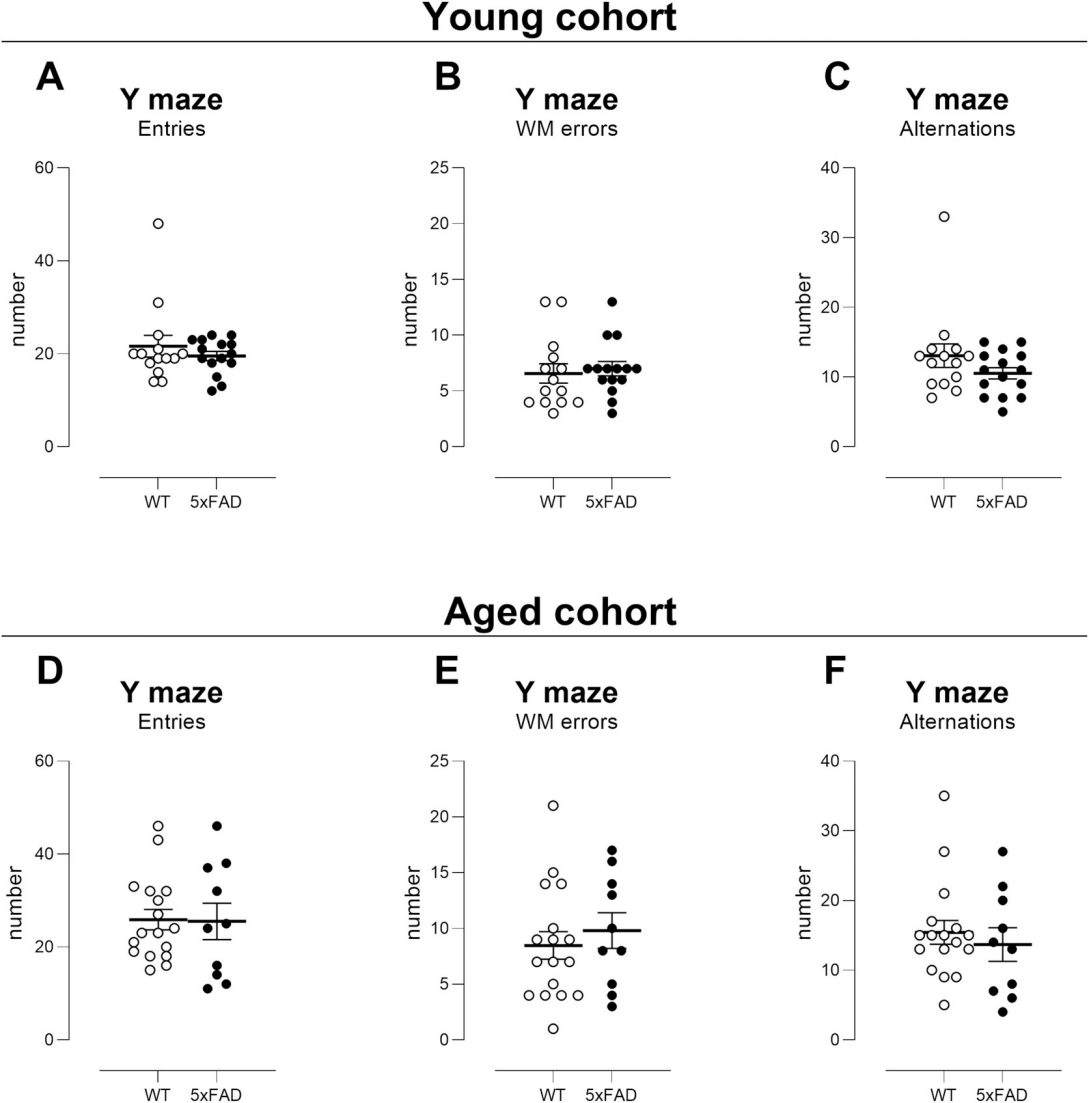
Spontaneous alternation is not impaired in 5xFAD TG mice. Deficits in spontaneous alternation in the Y maze were never observed, either in the Young cohort (tested for 5 minutes) or the Aged cohort (tested for 8 minutes). Videos were analysed manually by a blinded observer for number of entries (A, D: an entry was when the hindquarters entered an arm); unsuccessful triplets or working memory deficits (B, E: re-entries within a triplet) and successful alternations or triplets (C, F: e.g., ABC, CBA, ACB). One TG mouse from the Young cohort and two TG mice from the Aged cohort did not reach entry number threshold of 10 and were not included in analyses. Group sizes: Young, N = 14 WT, N = 15 TG; Aged, N = 17 WT, N = 10 TG. Symbols show data from individual mice with lines depicting mean ± sem.

Deficits in spontaneous alternation were detected in neither the Young nor Aged group (Fig 3A–3F; T-tests not significant (ns) for WT versus TG; 2-way ANOVA ns for genotype and ns for age x genotype interaction, no post-hoc tests were significant), showing that this outcome measurement is not optimal for therapeutic trials. Our data are in keeping with published data using large, balanced groups of young 5xFAD mice in this test [59]. We note that we used 5 minutes [39] for testing the Young cohort and 8 minutes [26, 44, 45] for testing the Aged cohort but neither revealed cognitive deficits. Thus, testing time is unlikely to have resulted in an inability to detect cognitive deficits but may have affected the number of entries made. We note that we also did not detect any deficits in spontaneous alternation in an additional, separate, smaller group of mice tested in pilot trials (4m old; female, N = 8 WT, N = 11 TG tested for 5 minutes). No power or sample size calculations were thus determined.

#### Forced alternation

Forced alternation was unsuccessful in examining cognitive performance as the majority of mice failed to reach the activity criterion (10 entries within 5 minutes: 50% of WT mice and 31% of TG mice reached criterion; unlikely to be related to anxiety as there was no change in occupancy of a central 20cm^2^ square in the open field: t test, T(24) = 1.9, ns). Moreover, the remaining WT mice did not show evidence of a preference for the novel arm (preference index one-sample T-test versus theoretical 0.5 preference index, not significant). An earlier pilot trial did reveal a preference in WT mice for the novel arm (preference index one-sample T-test versus theoretical 0.5 preference index, p<0.05) but 90% of WT mice reached entry criterion (9/10 mice). Thus, this test is highly dependent upon motor activity and results vary because of it. No power or sample size calculations were thus determined. This test was not examined in the Aged cohort.

#### Novel object recognition

**Fig 4:**
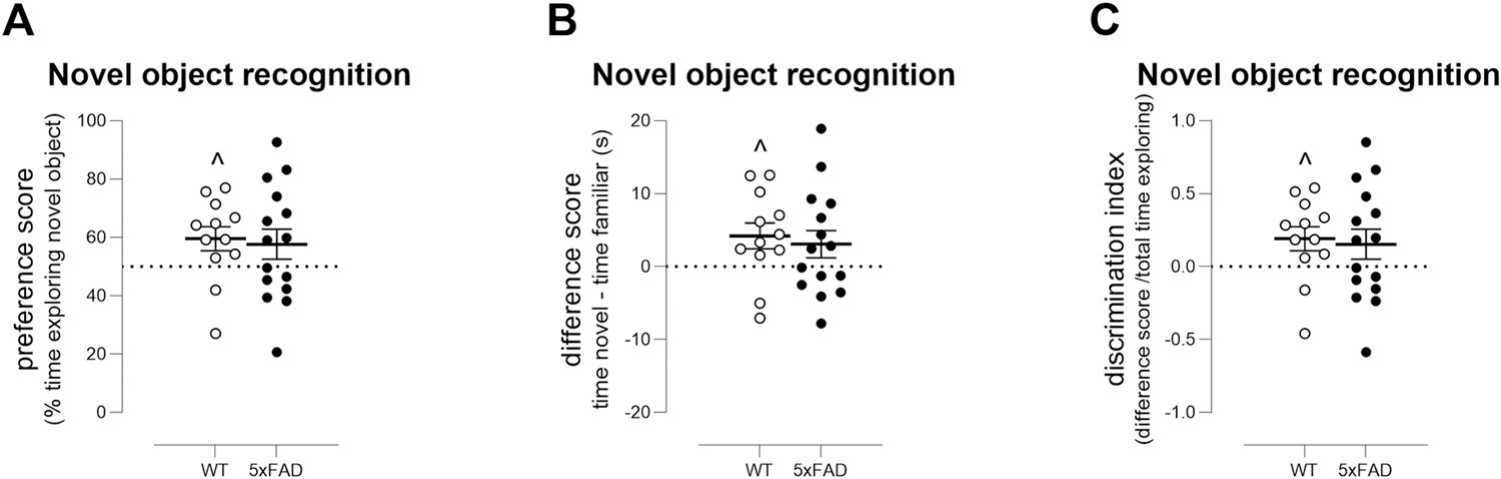
Novel object recognition is not a robust outcome measure for preclinical trials in 5xFAD mice. WT mice displayed a weak preference for the novel object based upon preference score (A), difference score (B) and discrimination index (C) (^p<0.05, one-sample T-test compared with theoretical values of 50% (A, dotted line), 0 (B, dotted line) and 0 (C dotted line)). TG mice did not show preference for the novel object (one-sample T-tests). However, there was no difference in performance between the genotypes (unpaired T-tests). Young cohort tested only: N = 12 WT mice and N = 15 TG mice as two WT mice and one TG mouse did not reach threshold for exploration during the familiarisation stage. Symbols show data from individual mice with lines depicting mean ± sem.

Mice were habituated to the test arena, and 24hrs later, they were familiarised to objects within the arena (familiarisation) and after 3hrs, they were tested for recognition of a novel object within the arena (novel object recognition). There was no difference between genotypes in exploration of the objects during the familiarisation stage, showing no intrinsic preference for either object set (effect of genotype: F(1,23) = 3.4, ns; genotype x object interaction: F(1,23) = 0.5, ns). During familiarisation, mice were required to reach a threshold of 20s exploration [47]: N = 2 WT and N = 1 TG did not reach threshold. All mice that achieved criterion were brought through to novel object recognition. During novel object recognition, 2 minutes testing time was not sufficient; however, all mice explored the objects within 5 minutes, similar to previous findings [47, 60]. WT mice showed a weak preference for the novel object (Fig 4A, WT preference score: p<0.05 versus a theoretical value of 50%) but TG mice did not. Similarly, WT mice showed novel object recognition based upon their difference score (Fig 4B, WT difference score: p<0.05 versus a theoretical value of 0) and upon discrimination index (Fig 4C, WT discrimination index: p<0.05 versus a theoretical value of 0); no such recognition was observed in TG mice. Nevertheless, this outcome measure was not robust, and T-tests did not reveal a difference between the genotypes: for difference scores, the post-hoc power was 6%, Cohen’s D ≅ 0.2 and the sample size required to detect a treatment effect equivalent of bringing TGs to WT performance was several hundred mice. This test was not examined in the Aged cohort.

#### Morris water maze: Young cohort

Cognitive tests that use swimming are immune to issues of motivation and spontaneous activity [33] that can be problematic in tests such as the Y maze, novel object recognition and forced alternation. The Morris water maze has been widely used in this line of mice [61].

**Fig 5:**
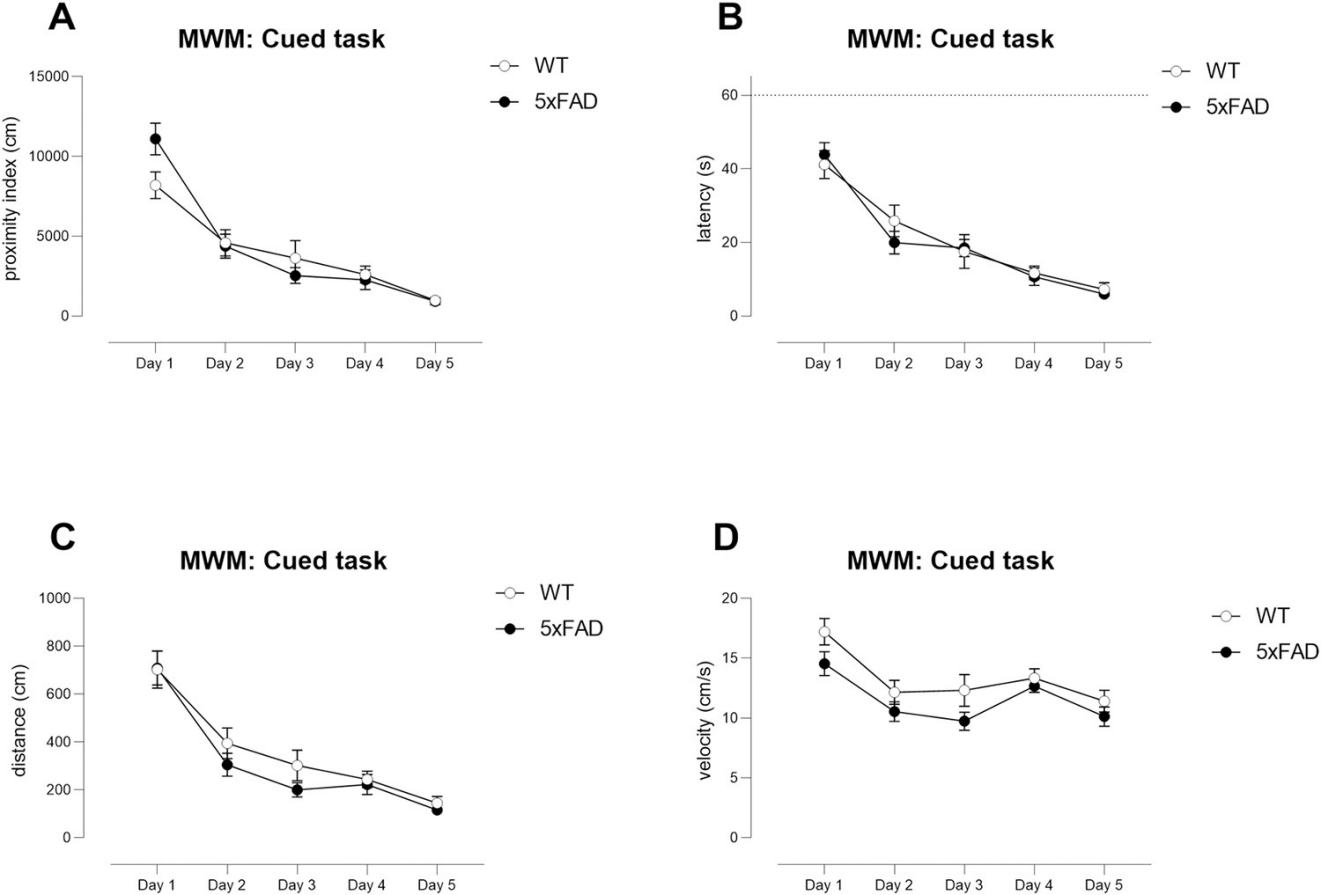
Cued learning in the Young cohort of WT and TG 5xFAD mice. As part of the Morris water maze testing protocol, mice were initially tested for their ability to find a submerged platform flagged with a highly salient flag (cue). No differences between genotypes were observed in latencies to find the platform (B) or in distance travelled (C) although the slope of learning over days, based upon Gallagher’s proximity (A), was slightly faster in TGs. Swimming speed (D) was similar between WTs and TGs. Young cohort: N = 13 WT, N = 16 TG. One WT mouse failed to learn despite additional guidance and was not analysed. Symbols show mean ± sem.

Using standard protocols [33], both WT and TG mice from the Young cohort learned to find the submerged, flagged (cued with a highly salient cue) platform. A difference in slope of learning over days was observed between genotypes when using proximity index, with TG mice displaying a slightly faster slope in learning (Fig 5A, WT versus TG, proximity: day x genotype interaction: F(4,108) = 3.98, p<0.01). However, at no point were there specific differences between genotypes on any specific day (Šídák’s multiple comparisons test, all ns). When using other typically used outcome measures, no differences between genotypes was observed (WT versus TG, day x genotype interaction: Fig 5B latency, F(4,108) = 1.040, ns; Fig 5C distance, day x genotype, F(4, 108) = 0.7, ns). Importantly, velocity was similar between genotypes (Fig 5D, day x genotype interaction: F(4,108) = 0.8, ns). An overall genotype effect was never observed (Gallagher’s proximity: F(1,27) = 0.1092, ns; latency, F(1,27) = 0.09, ns; distance: F(1,27) = 0.9, ns; velocity: F(1,27) = 3.3, ns). Thus, mice displaying appropriate visual acuity then continued onto spatial and reversal learning phases. No power analyses or group size calculations were performed using data from this task.

**Fig 6:**
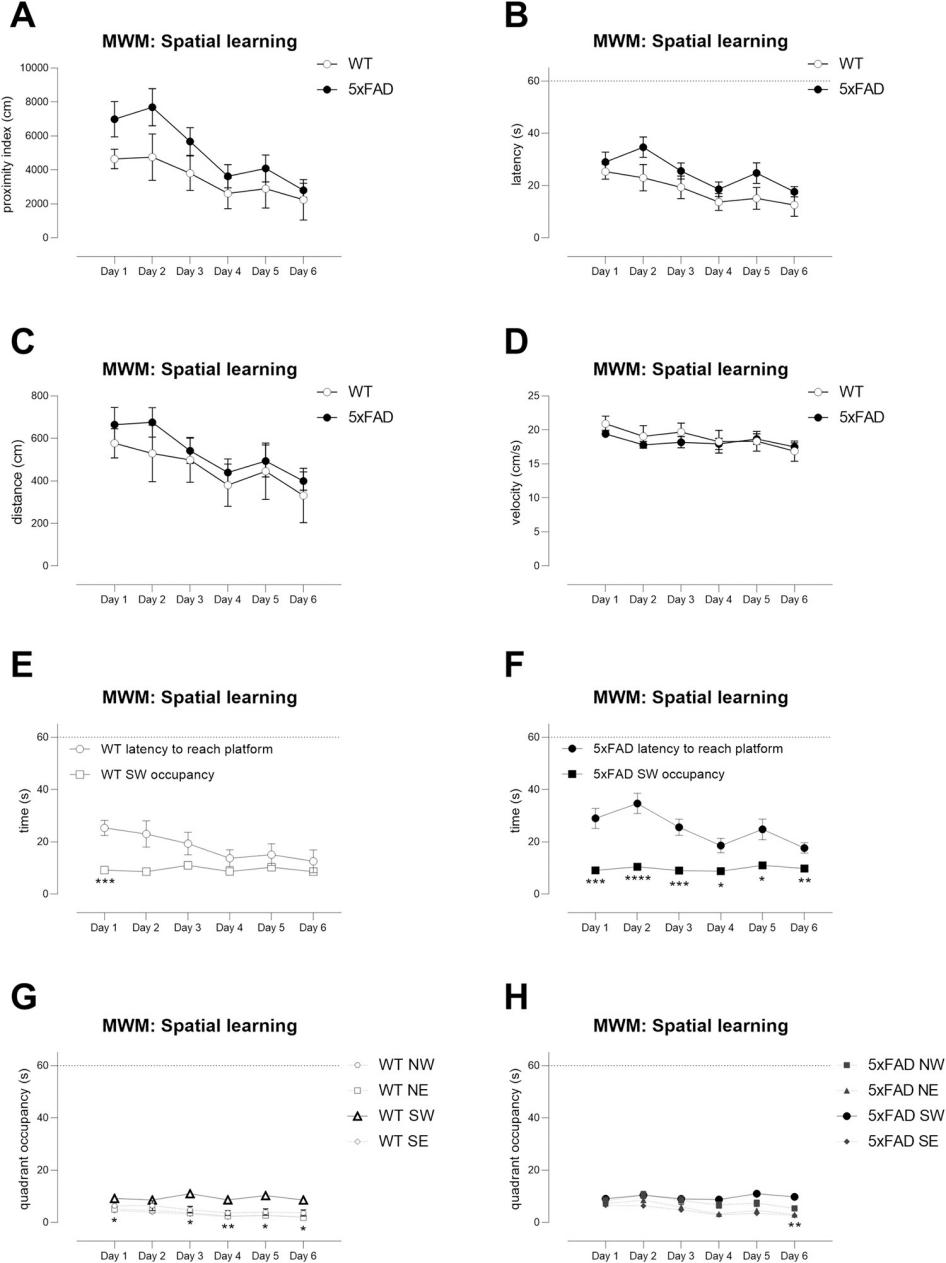
No major deficits in the Morris water maze spatial learning phase in the Young cohort of 5xFAD TG mice. During spatial learning in the MWM no major deficits were observed in learning in Young TG mice based upon Gallagher’s proximity (A), latency (B) and distance travelled (C). However, there was a slight impairment in efficiency of search strategy in TG mice, with WT mice showing a greater proportion of their time in the correct quadrant (SW quadrant) over time (E) and compared with other quadrants (G) compared with TG mice (F, H). Swimming speed was similar between the genotypes (D). E, F, asterisks indicate significant differences compared with same-genotype, same day data * p<0.05, ** p<0.01, ***p<0.001, ****p<0.0001. G, H: asterisks indicate differences in occupancy of SW quadrant versus other quadrants only when SW occupancy was significantly different to all three other quadrants * p<0.05, ** p<0.01, ***p<0.001, ****p<0.0001. N = 13 WT, N = 16 TG. Symbols show mean ± sem. Dotted lines on axes show maximum trial length of 60s.

During spatial learning, when the platform was submerged in the SW quadrant, TG mice showed no impairments in proximity, latency or distance travelled when compared with WT mice (Fig 6A–6C, WT versus TG, day x genotype; proximity, F(5,135) = 0.8 ns, latency, F(5,135) = 0.6 ns, distance, F(5,135) = 0.2, ns). However, search strategies used by TG mice were less efficient as WT mice latencies declined quickly over time to become largely reflective of the time spent in the correct quadrant whereas TG mice latencies did not (Fig 6E, WT, day x outcome measure: F(5,120) = 3.4, p<0.01; Fig 6F, TG, day x outcome measurement: F(5,150) = 4.4, p<0.001). This was revealed in more detail when quadrant occupancy per day was examined–WT mice tended to spend increasingly and exclusively more time in the SW quadrant (Fig 6G WT, effect of quadrant F(3,48) = 12.5, p<0.0001) whereas it was only on the final day of learning that TG mice spent the majority of their time in the SW quadrant (Fig 6H TG effect of quadrant F(3,60) = 16.3, p< 0.0001). Importantly, swimming speed was similar between groups (Fig 6D). Thus, the Young cohort show a very mild cognitive deficit in spatial learning, and sample sizes and power were not calculated.

**Fig 7:**
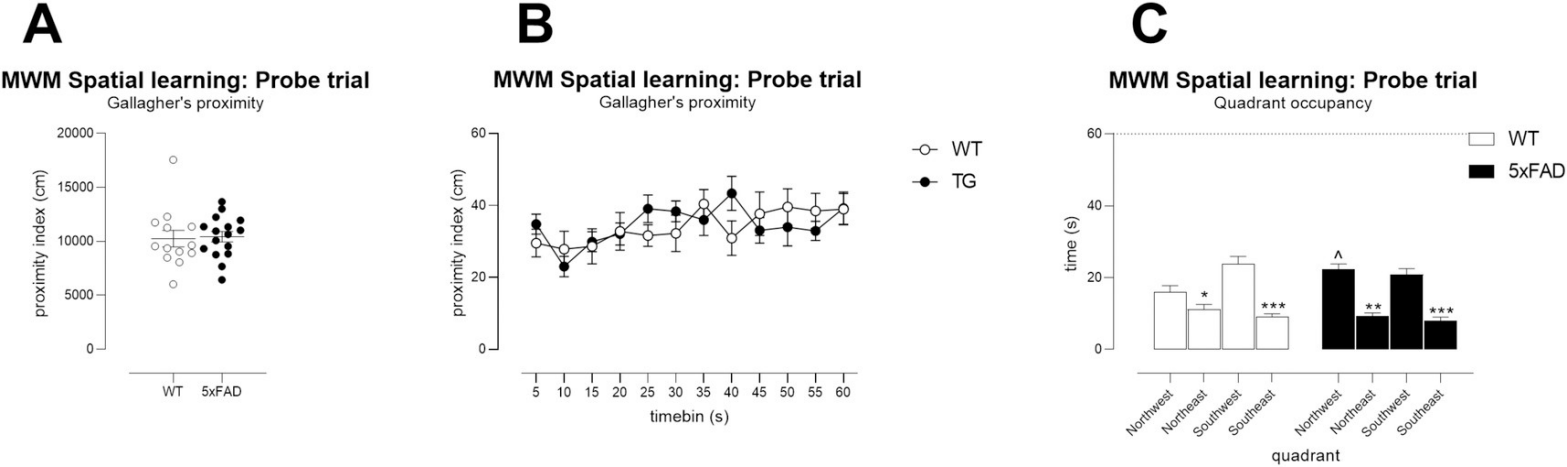
No major deficits in young 5xFAD TG memory, during probe testing following Morris water maze spatial learning. Memory of platform position following the spatial learning phase of the MWM (the probe trial). WT and TG mice from the Young cohort displayed similar memory for the platform position whether examined over the entire trial (Gallagher’s proximity per trial, A) or in 5-s intervals (Gallagher’s proximity per 5s, B). Quadrant occupancy of TG mice was slightly impaired compared with WT mice (C, genotype x quadrant F(3,81) = 3.306, p<0.05; ^ p<0.05 indicates a significant difference between TG and WT mice in the occupancy of NW quadrant; asterisks indicate significant differences compared with same-genotype occupancy of SW quadrant * p<0.05, ** p<0.01, ***p<0.001). This outcome measure does not reveal robust cognitive deficits in TG mice. N = 13 WT, N = 16 TG. A, Symbols show individual mice with lines showing mean ± sem. B, Symbols show mean ± sem. C, Columns show mean ± sem. Dotted lines on axes show maximum trial length of 60s.

Notably, when the platform was removed for the probe trial, TG showed a very similar performance to WT mice because Gallagher’s proximity, which is thought to be the most appropriate measure of performance during probe trials [62], showed no impaired performance in TG mice (Fig 7A and 7B). The quadrant preference of WT mice over the entire minute was slightly better than TG mice (Fig 7C, genotype x quadrant F(3,81) = 3.3, p<0.05) but both genotypes showed a relatively good preference for the SW quadrant. Power was not calculated, and sample sizes not determined.

**Fig 8:**
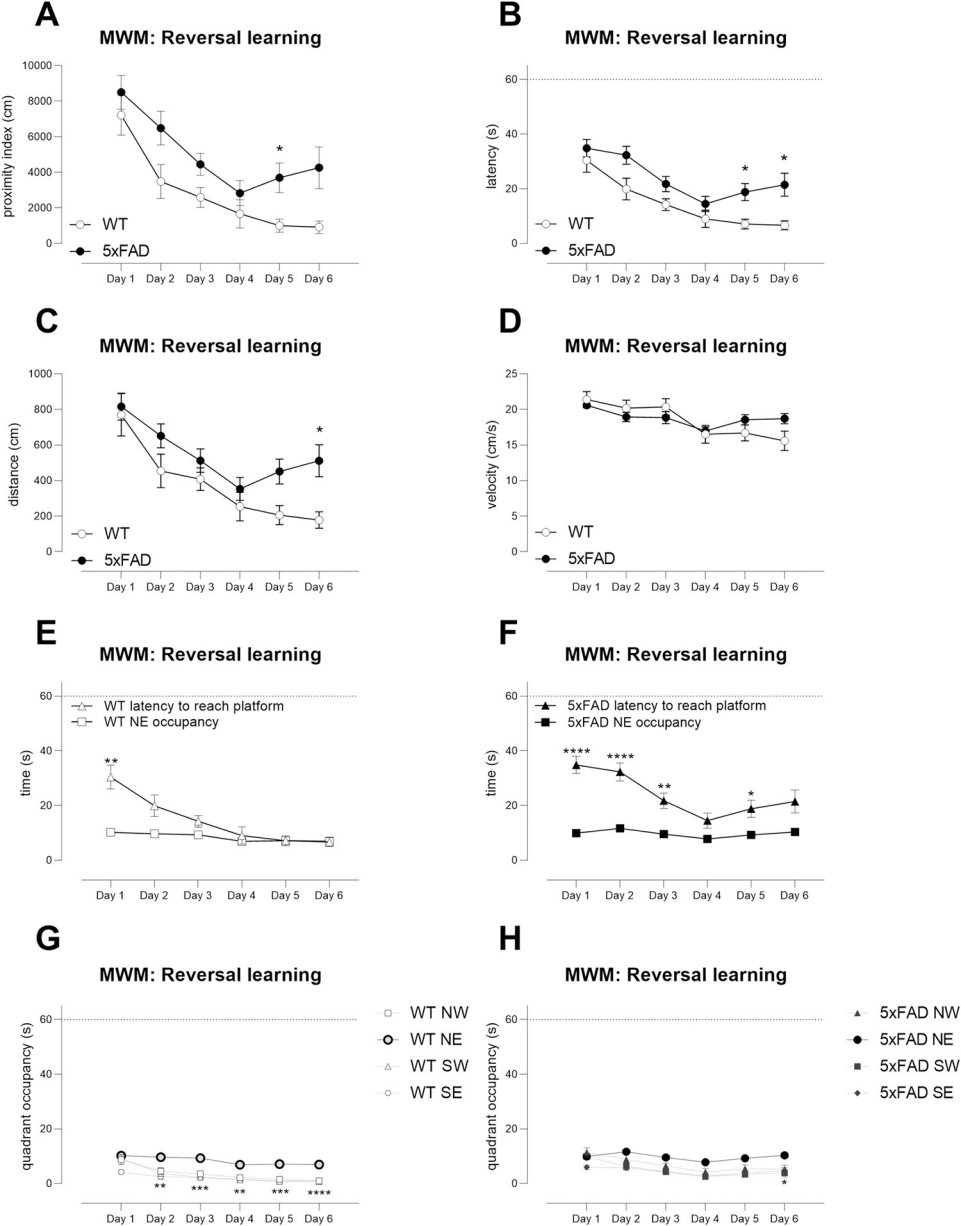
The reversal learning phase of Morris water maze testing reveals mild deficits in young 5xFAD TG mice. During reversal learning in the MWM, Young TG mice were impaired in learning the new platform position (NE) compared with WT mice (proximity, A; latency, B; distance, C). Swimming speeds were similar between genotypes (D). TG search strategies were less efficient compared with WT mice as shown by the time taken to reach the platform versus latencies (compare E (WT) with F, TG) and by quadrant occupancies (G, H). E, F, asterisks indicate significant differences compared with same-genotype, same-day data * p<0.05, ** p<0.01, ****p<0.0001. G, H: asterisks indicate differences in occupancy of NE quadrant versus other quadrants only when NE occupancy was significantly different to all three other quadrants * p<0.05, ** p<0.01, ***p<0.001, ****p<0.0001. N = 13 WT, N = 16 TG. Symbols show mean ± sem. Dotted lines on axes show maximum trial length of 60s.

Next, reversal learning was tested, in which the platform was submerged in the NE quadrant. During the learning phase, TG mice showed a deficit that was sufficiently large to reveal an overall genotype effect for proximity (Fig 8A), latency (Fig 8B) and distance travelled (Fig 8C) that was consistent over time as there was no genotype x day interaction (WT versus TG, effect of genotype: proximity F(1,27) = 8.2 p<0.01; latency, F(1,27) = 11.5, p<0.01; distance travelled, F(1,27) = 6.3, p<0.05; WT versus TG, day x genotype interaction: proximity F(5,135) = 0.8, ns; latency, F(5,135) = 1.1, ns; distance travelled, F(5,135) = 1.4, ns). The search strategy of WT mice was excellent, as their latencies essentially normalised to quadrant occupancy and was already statistically similar by D2 (Fig 8E) whereas TG mice took until D4 to normalise (Fig 8F). This was also revealed when examining quadrant occupancy–the occupancy of the NE quadrant was significantly different to all other quadrants by D2 in WT mice (Fig 8G), but this was not the case until the final day of training in the TG mice (Fig 8H).

**Fig 9:**
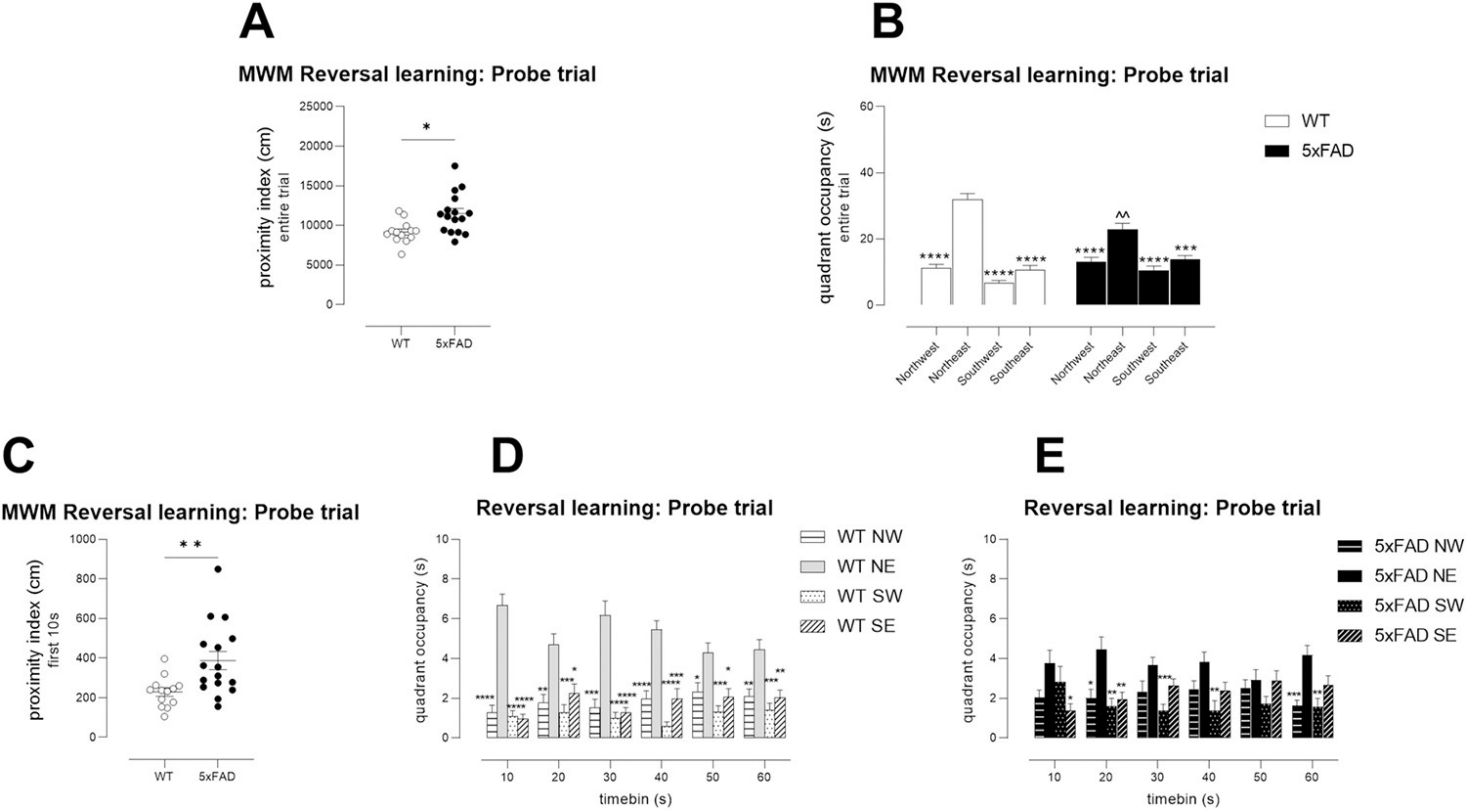
Mild memory deficits in Young 5xFAD TG mice during probe testing, following Morris water maze reversal learning. Young TG mice showed mildly impaired search strategies during probe testing following reversal learning in the MWM, as shown by increased proximities during the entire probe trial (A) or during the first 10s of the probe trial (C). TGs did prefer the correct quadrant (B) but their selection was less robust as shown by analysis of their quadrant occupancy over the length of probe trial (E) compared with WT mice (D). B, caps denote a significant difference between TG and WT mice in occupancy of the NE target quadrant ^^ p<0.01; asterisks depict significant differences compared with same-genotype NE occupancy ***p<0.001, ****p<0.0001. D, E asterisks depict significant differences compared with same-genotype NE occupancy per timepoint. * p<0.05, ** p<0.01, ***p<0.001, ****p<0.0001. N = 13 WT, N = 16 TG. A, C, Symbols show individual mice with lines depicting group mean ± sem. B, D, E, Columns show group means ± sem.

During the probe trial, the proximity index of TG mice was higher than in WT mice, indicating that they spent their trial at distances further from the learned platform position compared with their WT counterparts (Fig 9A, unpaired T-test). Similarly, although both genotypes displayed a clear preference for the NE quadrant, TG mice showed less robust preference (Fig 9B, quadrant x genotype interaction F(3,81) = 6.6, p<0.001). This was particularly obvious in early parts of the probe trial, when WT mice make the greatest effort to search near the supposed platform position (proximity for the first 10s, Fig 9C, WT versus TG p<0.01; Fig 9D and 9E: quadrant occupancy x time, WT time period x quadrant F(15,240) = 3.2, p<0.0001; TG time period x quadrant F(15,300) = 1.7, p<0.05). This is in keeping with the best performance of WT mice occurring during the first 10-15s of the probe trial [62]. Nevertheless, TG mice were not greatly impaired as they showed significantly greater occupancy of the NE quadrant compared with other quadrants.

Thus, TG mice from the Young cohort can learn 1) to find a cued platform, 2) to find a submerged platform and remember its position and 3) to find a submerged platform in a new position and remember its position. However, search strategies revealed mild deficits. We therefore calculated power and sample sizes based upon proximity index [62, 63] and quadrant occupancy from the probe trial of the reversal learning phase. Data from the entire reversal probe trial and the first 10s of the reversal probe trial were used (Table 3, N = 8–22 mice per group required to detect a treatment effect similar to bringing performance to WT levels with 80% power and α = 0.05). We note that variability of quadrant occupancies were lower and provided lower estimates of sample size. Nevertheless, proximity index is empirically the optimal measure of memory in MWM probe trials [62, 63].

#### Morris water maze: Aged cohort

**Fig 10:**
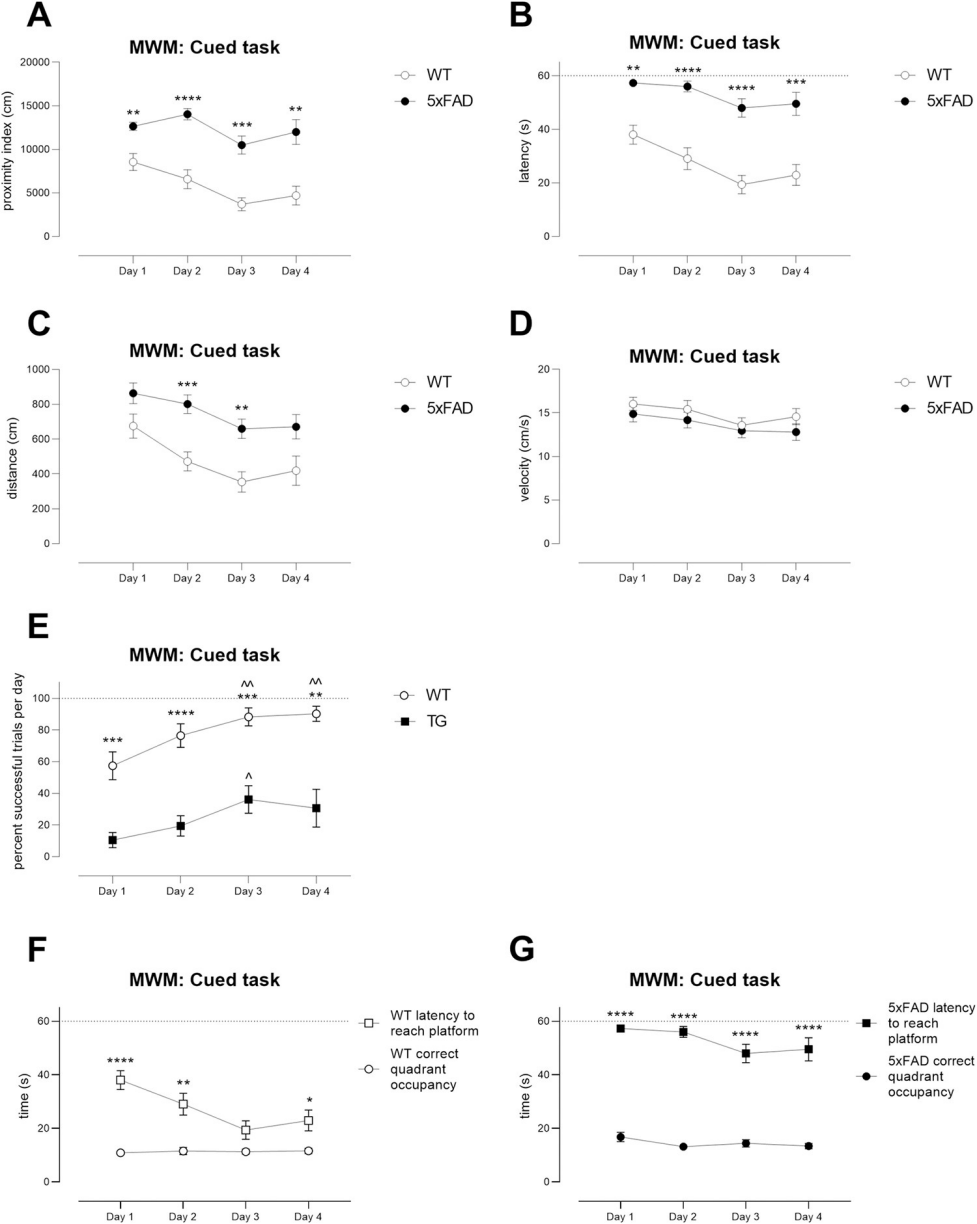
Major deficits during the cued learning phase of Morris water maze testing in Aged WT and TG 5xFAD mice. Performance in the MWM during cued learning, in the Aged cohort (A, Gallagher’s proximity; B, latency; C, distance travelled; D, swimming speed; E, percent successful trials per day). Aged TG mice are greatly impaired in this task, precluding the ability to test spatial or reversal learning or memory. Although WT mice latencies tended to decline to the time spent in the correct quadrant (F), no such pattern was observed in TG mice (G). Note that all mice were Pde6brd1 wildtype or heterozygous. A-E: asterisks depict statistical differences between the genotypes on the days shown * p<0.05, ** p<0.01, ***p<0.001, ****p<0.0001. E, caps depict differences compared with day 1, ^ p<0.05, ^^ p<0.01. F, G: asterisks depict statistical differences within genotypes and between outcome measures, on the days shown * p<0.05, ** p<0.01, ****p<0.0001. N = 17 WT, N = 12 TG. Symbols show group means ± sem. Dotted lines on axes show maximum trial length of 60s.

**Fig 11:**
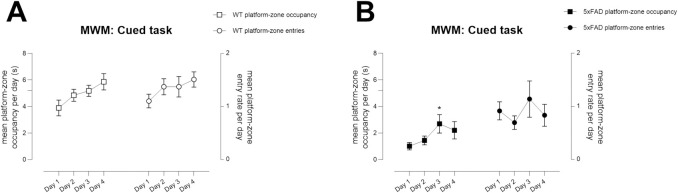
Failure to learn to recognise the platform as an escape in Aged 5xFAD TG mice during the cued learning phase of Morris water maze testing. Platform-zone occupancy (see left Y-axes) and crossings (see right Y-axes) in Aged WT (A) and TG (B) mice during the Cued (visual) phase of MWM testing, where the platform is flagged with a highly salient cue. A, WT mice showed excellent learning over time and learned to stay on the platform, which was associated with increased success rate as shown in Fig 10E. B, Although TG mice entered the platform zone, which was larger than the platform itself to accommodate platform-clinging that was common with this group, they failed to recognise the platform as an escape and thus, showed reduced time in the platform zone compared with WTs. * p<0.05 compared with Day 1, same genotype, same outcome measure. N = 17 WT, N = 12 TG. Symbols show group mean ± sem. Additional tick on left Y-axis denotes the time required to spend on platform (5.2s).

In great contrast, the performance of TG mice from the Aged cohort was profoundly impaired (Fig 10). We did not perform spatial learning or reversal learning due to the robust deficits displayed by TG mice in this group during cued (non-spatial) testing. We adjusted several aspects of our protocol as the TG mice were frail by this age, including warming the water to 23–24°C (from 22°C [33]), providing a much longer inter-trial interval (60 minutes, from 10–15 minutes), reducing number of trials per day (from 4 to 3), lowering the platform height to enable the mice to climb on (~2cm below surface, from ~1cm below surface for the Young cohort) and reducing number of test days (from 5 to 4). WT mice were capable of consistently finding the cued platform by day 3 and they improved greatly over time (Fig 10A, 1-way ANOVA of WT proximity index, F(2.3, 36.5) = 12, p<0.0001; D1 versus D3 p<0.0001; D1 versus D4 p<0.001, D2 versus D3 p<0.02). In contrast, there was no overall improvement in performance in TG mice (Fig 10A, 1-way ANOVA of TG proximity index, F(1.7, 18.4) = 3.5, ns). Indeed, by day 4, only about 31% of trials were successful in the TG group (Fig 10E). When analysing proximity to include genotype as a factor, there was a large overall effect of genotype (Fig 10A and 10F(1,27) = 32.3, p<0.0001) and WT performance was superior to TG on all days (Fig 10A). Latencies and distance travelled also showed greatly impaired learning in the TG mice (Fig 10B and 10C; S2 Fig in S1 File; Latency effect of genotype F(1,27) = 35.3, p<0.0001; Distance effect of genotype F(1,27) = 11.5, p<0.0001) although swimming speed was similar (Fig 10D, effect of genotype F(1,27) = 1.2, ns, genotype x day interaction F(3,81) = 0.3, ns). As with the Young cohort, latencies of WT mice tended to decline to the time spent in the correct quadrant (Fig 10F; day x outcome measure F(3,96) = 11.5, p<0.0001), whereas no such pattern was observed in the TG group (Fig 10G; day x outcome measure F(3,66) = 2.6, ns). Although TG mice consistently entered the platform zone, which was larger than the platform itself to accommodate platform-clinging that was prevalent in this group (Fig 11B), time spent in the platform zone was reduced in comparison to WT mice and they showed little improvement over time (Fig 11; time in platform zone: effect of genotype F(1,27) = 31.3, p<0.0001; platform crossings: effect of genotype F(1,27) = 9.3, p<0.01). Moreover, we noted that although both genotypes “bumped” the platform without getting onto it during the early part of training, over successive days of testing the WTs learned to get on the platform and escape whereas the TG mice did not (final day of testing, mean number of trials showing platform bumping per mouse, Mann-Whitney U test, WT versus TG, p<0.05). Consequently, TG mice spent more time in non-platform quadrants than WT mice (effect of genotype, F(1,27) = 29.6 p<0.0001; day x genotype F(3,81) = 4.3 p<0.01).

Previous data has suggested severe deficits during all stages of MWM in 5xFAD TG mice at this age including during visual learning [64], which likely confounds any spatial or reversal learning at this age. Moreover, pigmentation has been suggested to impact performance in the MWM [64]. Although we noted a general effect of coat colour in WT mice but not in TG mice, we are cautious of these results given the small group sizes involved for some of the coat colours, which are similar to those used in the paper quoted (coat colour used as a surrogate for albinism or pink-eye dilution [64]; our groups: WT N = 12 brown or black fur, N = 5 white fur; TG N = 5 brown or black fur, N = 7 white, beige or grey fur). Furthermore, white beige or grey WT mice easily outperformed white, grey or beige TG mice (mice with white, grey or beige fur only, effect of genotype F(1,10) = 9.7, P<0.02) and there were no differences in daily performances between WT subgroups (white, grey or beige versus black or brown).

For power and sample size estimations for the Aged TG mice, the mean group proximity indices on day 4 of testing at 13-14m were used, which showed very high effect size (Table 2). Due to the large difference between genotypes, very small group sizes would be required to detect a treatment effect similar to bringing performance to WT levels (Table 3, N = 6–9) but for a smaller effect size to 30% improvement, group size estimates were 17–31, depending upon algorithm used to calculate sample size (Table 4).

**Table 4:** Group sizes required to detect a difference of 30% in TG mice in neuropathological or behavioural outcomes (only possible where difference between WTs and TGs was greater than 30%).

Outcome measure	Group size required to detect improvement of 30%^a^ in TG mice α = 0.05 80% power Group sizes 1:1			
**Neuropathological outcome measures**				
	Matlab	ClinCalc	Biomath	G-Power
Plaque load: Congo red-positive plaque density in frontal cortex at 4m	12	19	20	20
Plaque load: Congo red-positive plaque density in CA2-3 at 4m	12	18	20	20
Plaque load: Congo red-positive plaque density in dentate gyrus at 4m	7	8	10	10
Plaque load: Congo red-positive plaque density in subiculum at 4m	6	7	9	9
Microgliosis: IBA1-positive particle size in frontal cortex at 4m	6	8	9	9
Microgliosis: IBA1-positive particle size in subiculum at 4m	16	10	17	17
Astrogliosis: GFAP-positive percent area in frontal cortex at 4m	12	20	21	21
Astrogliosis: GFAP-positive percent area in in subiculum at 4m	4	3	<6	5
**Behavioural or general health outcome measures**				
MWM, Cued learning proximity index on Day 4: Aged cohort, to bring to level of WT mice	17	30	31	31

^a^Calculated only where differences between WTs and TGs was greater than 30%, for e.g., body weight in TG mice at 1 year of age was different from WTs by only 31% and so was not calculated for this table as it is essentially the same as in Table 3.

Group size estimates to enable use of a T-test to detect a 30% improvement in TG outcome: Mathworks: [52], ClinCalc [53], Biomath [54], G-Power [55].

Thus, up to 5–6 months, TG mice are capable of learning to find a platform and remembering its position, although search strategy is slightly impaired relative to WT mice, in keeping with previous data [64]. By 13 months, TG mice are profoundly impaired and cannot learn to consistently find a cued platform in the Morris water maze, precluding the ability to determine spatial or reversal learning or memory. We note that all mice (Young and Aged cohorts) were genotyped for the Pde6brd1 mutation that is common in these mice and only wildtype or heterozygous mice were used for testing [42].

### Neuropathological endpoints

#### Plaques: Congo red and Fluorojade C

**Fig 12:**
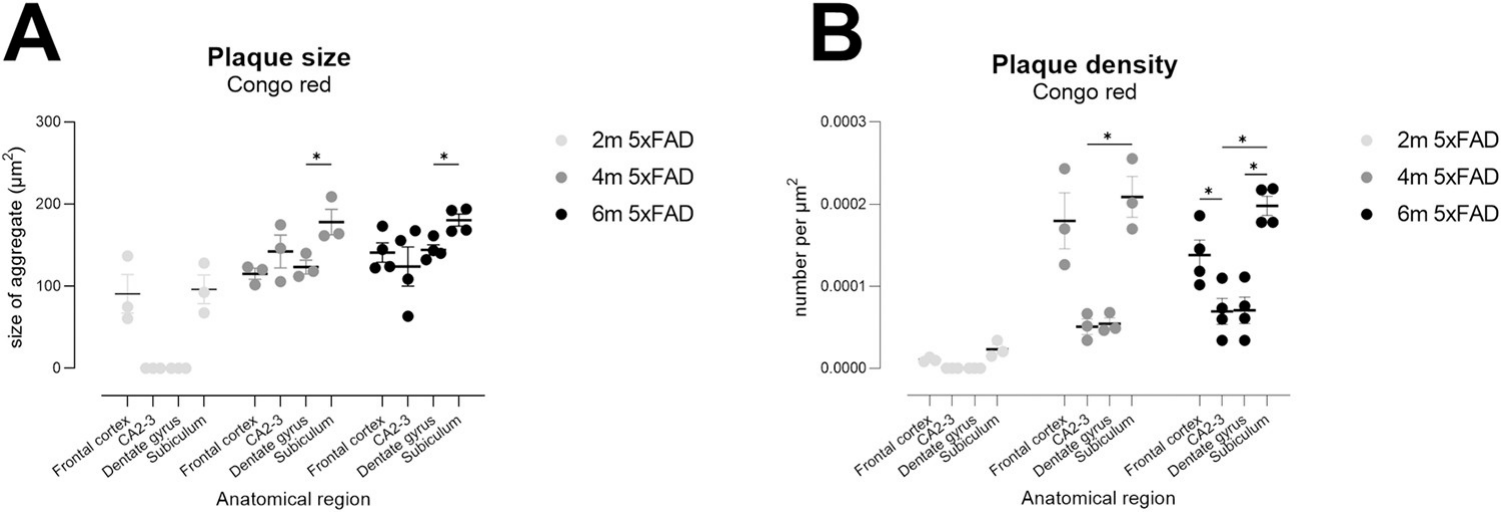
Congo-red plaque density is a suitable outcome measure for preclinical trials in 5xFAD TG mice. Congo-red-positive amyloid plaques in 2m- to 6m-old 5xFAD TG mice. Measurements of plaque size (A) and plaque density (B) suggest that density is a more robust measure and that it provides a more dynamic range for analysis of potential treatment effects, likely due to the low number of plaques present in 2m-old TG mice. Data points show data from individual mice (N = 3–4 female TG mice per timepoint) with lines showing mean ± sem. Asterisks depict within-age statistical differences between regions, * p<0.05. fCtx, frontal cortex; DG, dentate gyrus.

Amyloid plaques are well known to develop over time in the 5xFAD mouse and first appear between 1 and 2 months of age [26]. Congo red birefringence is used in the clinic to identify neuritic plaques, a defining neuropathological feature of AD [65], and many Abeta aggregates are birefringent in these mice [66, 67]. Here, we analysed plaque sizes and density in subiculum (Sub), CA2/3 and dentate gyrus (DG) of the hippocampus and in the frontal somatomotor cortex (fSMctx) to provide estimations of appropriate group sizes for use in preclinical trials. As expected, plaque density increased over time (Fig 12B; effect of age F(2,7) = 30.6, p<0.001), with Sub and fSMctx showing a greater slope than DG and CA2/3 (Fig 12B; anatomical area x age, F(6,21) = 8.1, p<0.0001). Plaque size also increased with age, with subiculum tending to develop the largest plaques (Fig 12A effect of age, F(2.1, 14.6) = 17.9, p<0.001; age x region interaction, F(6,21) = 5.2, p<0.01) although the slope in increase of size was slower than the slope in increase in density (Fig 12A versus B, respectively), thus revealing density to be a more robust outcome measure.

Fluorojade C has been used as an indicator of degenerating neurons but is known as a marker of amyloid plaques [68, 69]. This marker provided more variable data and less robust changes with increasing age (effect of age upon plaque density F(2,7) = 9.2, p<0.05; anatomical area x age interaction F(4,14) = 1, ns). Given the well-known increase in amyloid in these mice over the ages examined here (2m – 6m), these data suggest that Congo red may be a more valuable and less variable endpoint measurement. Showing the robustness of Congo-red plaque density outcome measures (Table 2), small sample sizes (N = 1 to <6) would be required to detect a treatment effect similar to bringing plaque density of 4m-old TG mice to that of 2m-old mice (Table 3) whereas group sizes of 6–20, depending upon region and algorithm, would be required for a smaller effect size of 30% improvement (Table 4).

#### Microgliosis

**Fig 13:**
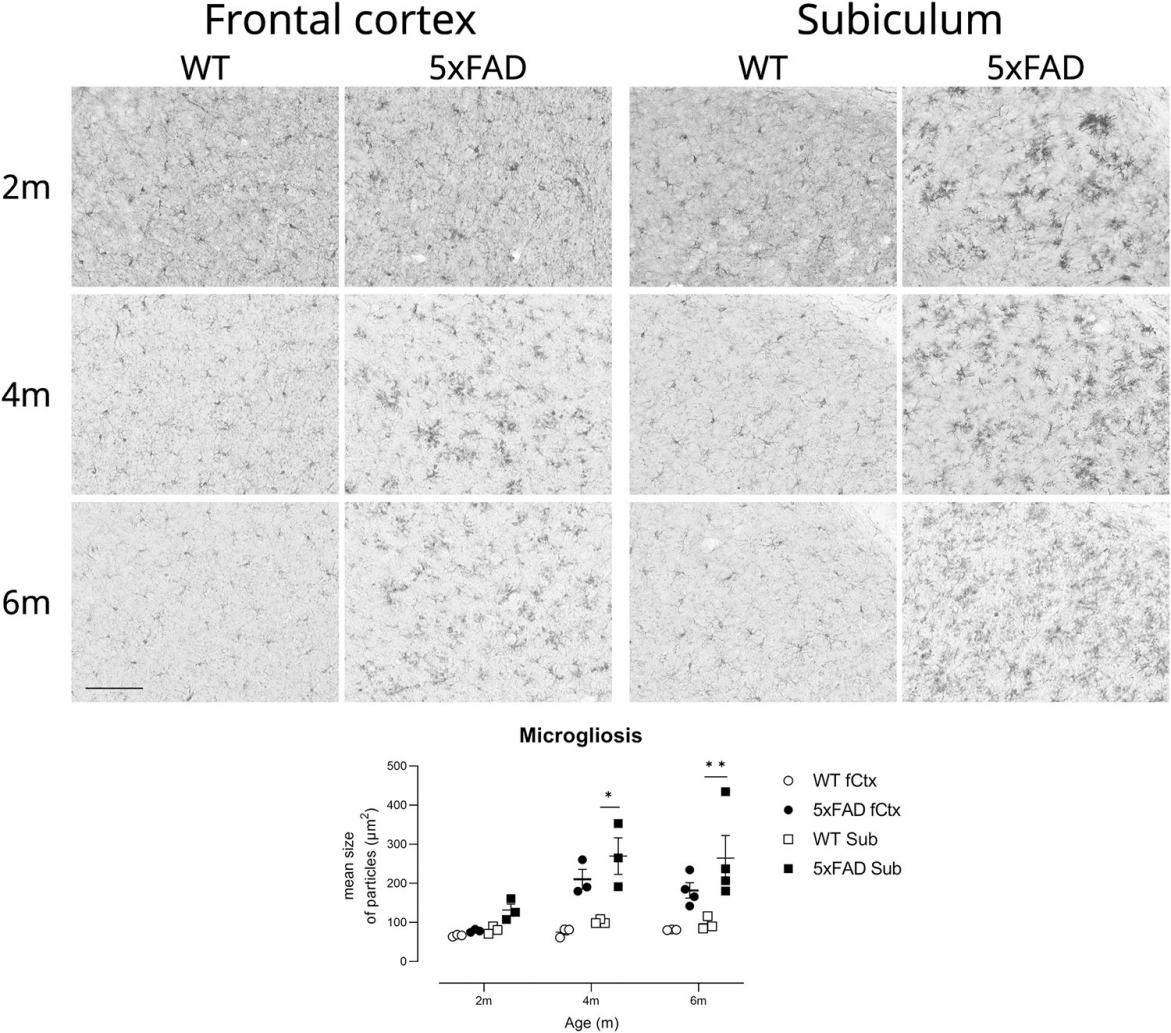
Subiculum microgliosis provides the highest power to detect treatment effects on microgliosis in 5xFAD TG mice. Microgliosis in 5xFAD mice develops with age and is well known and well-characterised. Photomicrographs show exemplar images from sagittal sections of layers V-VIa of frontal cortex and from subiculum, corpus callosum can be seen at the top right of each image of the subiculum. As shown in Table 2, differences between genotypes provides high power and low group sizes to detect a treatment effect equivalent to normalisation to WT levels in frontal cortex and subiculum. However, the difference between genotypes in frontal cortex did not survive post-hoc analysis in the context of a three-way ANOVA examining genotype, age and region. Thus, the most robust statistical power with these group sizes was observed in subiculum. Asterisks depict within-age statistical differences between genotypes, * p<0.05, ** p<0.01. Scalebar in lower left = 100μm, for all photomicrographs. Symbols show individual mice with lines depicting group means ± sem. fCtx, frontal cortex; Sub, subiculum.

Many authors have proposed analysis methods for quantification of morphology of microglia [34, 70–72]. However, analysis of morphology is labour intensive, limiting the numbers of individual cells that can be quantified. Here, we quantified area covered by IBA1 antibody staining based upon particle analysis following batch processing in ImageJ [34], which provided a simple, semi-automated and relatively efficient method of measuring microglia. We examined frontal cortex (somatomotor and somatosensory) and subiculum (Fig 13). Using this method, we were able to efficiently quantify microglia, with high power and large effect sizes (Table 2) to enable detection of treatment effects. As expected, there was an overall effect of anatomical area (F(1,13) = 6.6, p<0.05) and age (F(2,13) = 8.4, p<0.01) and a highly significant overall effect of genotype (F(1,13) = 43.1, p<0.0001). The slope of increase in particle size caused a significant interaction of age with genotype (F(2,13) = 5.3, p<0.05) although we did not detect an overall interaction of age with anatomical area and genotype (F(2,13) = 0.1, ns). The robust differences in subiculum compared with WT mice survived post-hoc testing following a three-way ANOVA, showing its well-known vulnerability to microgliosis in these mice. Nevertheless, we obtained estimates of sample sizes and power based upon data from subiculum and from frontal cortex as T-tests involving these areas may be appropriate for some preclinical trials. Group sizes of 2–9, depending upon algorithm and region, were estimated to be sufficient to detect a change in microgliosis equivalent to normalisation to WT levels (Table 3) and with estimates of 6–17 for an improvement 30% (Table 4).

#### Astrocytosis

**Fig 14:**
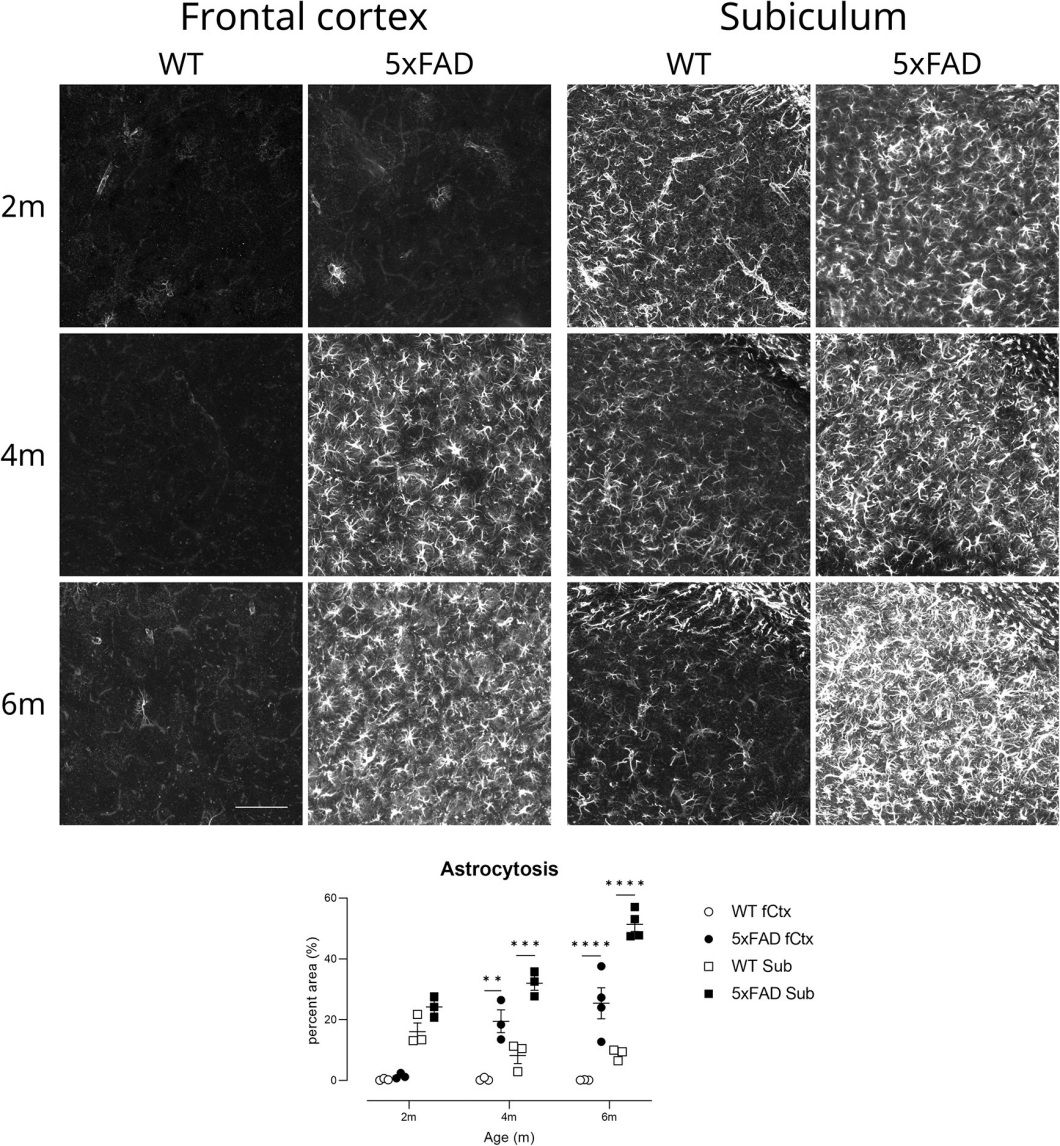
Both frontal cortex and subiculum provide sufficient power to detect treatment effects on astrocytosis, from 4 months in 5xFAD TG mice. As expected, and has been well characterised, astrocytosis in 5xFAD mice developed with age, particularly in frontal cortex and subiculum. Photomicrographs depict exemplar images from sagittal sections, lateral frontal cortex (layers VIa-V; left) and subiculum (right). The corpus callosum can be seen at the top right of each image of the subiculum. Graph: Large differences between genotypes were observed: asterisks depict within-age statistical differences in percent GFAP-stained area between WT and TG mice, ** p<0.01 *** p<0.001, **** p<0.0001. Scalebar in lower left = 100μm, for all photomicrographs. Symbols show individual mice with lines depicting group means ± sem. fCtx, frontal cortex; Sub, subiculum.

Astrocytes are normally absent from cortex in young mice [28] but although normally present in subiculum, a simple outcome measure of percent area of GFAP-positive staining was sufficient to provide excellent power to detect treatment effects in both anatomical areas (Fig 14). Astrocyte density increased over time in the TG mice, as previously demonstrated [26] (effect of genotype (F(1,13) = 138.8, p<0.0001) and worsened with age (age x genotype F(2,13) = 25.48, p<0.0001). Very different prevalences per anatomical area was noted (effect of anatomical area (F(1,13) = 109.7 p<0.0001; anatomical area x genotype (F(1,13) = 10.67, p<0.01) but as with microgliosis, the slopes were not sufficient to reveal an age x anatomical area x genotype interaction (F(2,13) = 1.825, ns). Again, using simple analysis methods, these robust outcome measures provided large effect sizes. The low within-group variabilities enabled estimates of very small groups of TG mice (N = <6) to detect normalisation of astrocytosis to WT levels at 4m and at 6m (Table 3) with estimates of 3–21 for smaller improvements of 30% (Table 4). Moreover, within-age comparisons between WT and TG mice survived three-way ANOVA and post-hoc testing.

## Discussion

The purpose of our paper was to examine sample size and effect size of behavioural tests that are commonly used in the 5xFAD line of mice for preclinical trials, as recommended by ARRIVE guidelines [40]. 5xFAD preclinical trials typically used young mice, and we were surprised by the mildness of cognitive impairment in the 5xFAD TG mice at ages up to 6m, despite the widespread use of young 5xFAD TG mice in preclinical trials using cognition as an endpoint measure. However, we note that authors are reporting increasingly a lack of cognitive impairment in these mice at these young ages [37, 38, 64]. Cognitive deficits are not robust until much later in life [64], and are accompanied by a late-developing hyperactive phenotype and a failure to gain weight [37, 56], all of which we replicate here. These more recent data, together with our own data shown here, may provide a more reproducible pattern of behavioural deficits in 5xFAD mice and appropriate group sizes.

We observed a mild deficit in learning the position of a submerged platform in the MWM during acquisition and reversal in Young TG mice (5m), as their quadrant occupancies revealed a less-efficient search strategy. Memory for the platform position showed mild impairments during acquisition stage. During reversal probe testing, although the TG mice chose the correct quadrant, proximity measures and quadrant occupancy again revealed mild deficits, in keeping with previous data [64].

In contrast, the majority of Aged TG mice did not show learning during the cued (non-spatial) task of the Morris water maze, which precluded our ability to subsequently complete spatial or reversal learning at this age. Proximity index to platform, which is likely to best represent spatial learning [33, 63], was deficient during learning and distances swam were longer than in WT mice. Motor performance itself was unlikely to affect performance as tasks reliant upon swimming are less impacted by changes in activity [33, 73] and also swimming speeds were similar between genotypes, although 5xFAD TG mice were frail at this age. Albinism or pink-eye dilution may affect MWM performance [64]. However, there was no difference in performance of TG mice when subgroups divided by coat colour were compared (coat colour was used as a surrogate for albinism or pink-eye dilution groups). Thus, genotype of 5xFAD TG mice is the determinant of behavioural deficits in the Morris water maze in Aged mice and other tasks. Pigmentation played a very minor role (S1 Table). Cued learning during MWM is thought to be simpler than spatial learning and to be egocentric in nature, and it can reveal deficits in sensorimotor function or a failure to recognise an “escape” [33]. We note that mice were Pde6brd1 wildtype or heterozygous [42]. However, although they entered the platform “zone”, TG mice did not achieve the same success rate as WT mice–they did not recognise the “escape”. WTs learned to get on the platform and escape whereas the TG mice did not, as they bumped into the platform and moved away more frequently than WT mice by the final day of testing.

Dorsal striatal lesions have long been associated with deficits in the visual, cued task of the MWM as the striatum is thought to play a major role in goal-directed navigation as opposed to the major role the hippocampus plays in spatial navigation that relies on distal cues ([74], reviewed in [75]). Moreover, head-direction cells, critical to all parts of MWM learning, and without which egocentric learning cannot take place, are found in many different regions of the brain, including dorsal striatum [73]. Although the striatum is not widely studied in these mice, striatal deposits of Abeta are observed with aging in these mice [37], and PET imaging revealed reduced levels of D2 receptor [76] and mGluR5 [77] in striatum in 9m-old 5xFAD TG mice. Intriguingly, patients with early-onset (63±4 yrs) Alzheimer’s disease and patients with AD (77±7yrs) show volume changes in dorsal striatum, specifically putamen [78, 79], which correlated with cognitive impairment [78]. Moreover, greater amyloid load in anterior and posterior putamen correlated with greater frailty in the elderly [80]. Finally, both egocentric and allocentric learning are impaired in AD patients and in individuals with amnestic MCI (reviewed in [81, 82]).

With respect to other cognitive tasks, a simpler test of working memory, that of spontaneous alternation, was never successful in showing impairments as we never observed deficits in Young or Aged 5xFAD TG mice. This is in keeping with other authors using large group sizes balanced for sex and for genotype [37]. Additional tasks that we examined included novel objection recognition, which did not reveal sufficiently robust cognitive deficits to warrant its use in preclinical trials, and forced alternation. Novel object recognition (NOR) was also hampered by a weak WT performance. Variability in this task (NOR) is well characterised, and it has been postulated that the task may be more robust for mice of the age used here, if initial exploration bouts of up to 20s, only, are examined [60]. However, this may not be the issue here as mean total exploratory times during our testing phase were ~22±2s for WTs and ~18±2s for TGs (mean ± sem, not significant). We note that all mice explored their objects for more than 20s during the familiarisation phase, which was our threshold for continuing with testing in the NOR [32, 47]. In the case of forced alternation in the T maze, this task was greatly affected by motivation to explore the arena. An earlier pilot trial showed sufficient mice achieving threshold for activity but in this cohort of mice, 50% of WT mice failed to reach threshold activity levels and indeed, almost 70% of TG mice failed to reach activity threshold. Given the lack of efficacy, we did not examine NOR or forced alternation in the Aged cohort but do report them here for transparency [17].

With respect to general health and activity, we noted hyperactivity in our Aged TG mice (tested between 13 and 14m) and that the TG mice showed reduced weight in comparison to WTs from as early as 4m of age. For analysis of body weight, we combined our groups (Young and Aged cohorts) and as such it is possible that these large group sizes enabled a significant difference between genotypes from an earlier age than previously reported [37, 56]. Nevertheless, weight loss in AD mice is not unexpected as weight loss is well known to progress with disease progression in human patients with AD [83, 84] and in individuals at risk for AD (A+T+), potentially downstream of Abeta deposition [85]. With respect to activity, it is possible that circadian rhythm alteration played a role in the hyperactivity that we observed in Aged TG mice. AD patients are well known to display alterations in circadian rhythm and in severe disease may benefit from light therapy [86–88] but evidence of circadian rhythm impairment in 5xFAD TG mice is inconsistent [37, 89].

In regard to behavioural and general health outcomes, the largest effect sizes between similarly aged WT and TG mice and the highest power to detect treatment effects were observed using data from spontaneous activity in a novel environment, body weight and visual learning in the Morris water maze in Aged TG mice, at approximately 1 year after first plaques and initial inflammation are observed in brain in these mice. Our neuropathological outcomes were very representative of previous data and we find that the density of Congo-red-stained plaques is very consistent, allowing small-group-size use in preclinical trials. Congo red is well known and used in the clinic for the identification of neuritic plaques due to its birefringence when bound to beta-pleated sheets [65]. Inflammation in the form of microgliosis and astrogliosis are also well known and characterised in AD patient brains and in these mice. Using published, relatively simple and efficient analysis methods, GFAP and IBA1 staining provided statistically powerful outcome measures to enable the use of small group sizes in preclinical trials, although as expected, the estimates of sample sizes varied with expected effect size (Table 3 versus Table 4).

Cohen’s D is a typically used measure of effect size, with 0.2, 0.5 and 0.8 generally indicating small, medium and large effect sizes, respectively [90]; however, in preclinical studies these boundaries are less useful [19], and indeed, here we show very large effect sizes for behavioural outcomes(Table 2). Neuropathological outcomes revealed extremely large effect sizes, and although there may be some inflation of effect size due to the small group sizes used [58], the large pre-existing literature supports these effect sizes. Moreover, we show clearly that group size varies with expected effect size, and for an unknown agent, small effect sizes would require large groups [19]. In reference to our power calculations, power is difficult to determine in advance of obtaining data [25]; and post-hoc power can be problematic as the null hypothesis may be correct even in the context of high post-hoc power [58]. However, given previous data showing the development of weight loss, hyperactivity and late-onset severe cognitive impairment in 5xFAD TG mice [37, 64] and the development of their neuropathology [26], the risk of the null hypothesis (that there is no difference between the genotypes) is greatly reduced, again, supporting the high power that we report for our outcomes.

Factors proposed to contribute to failures of clinical trials include methodological issues [91], patients having progressed too far by the time they have been treated [91, 92] or target selection [91, 93]. With respect to preclinical trials, a general lack of sample-size calculations has been noted as a serious issue in many preclinical trials [16]. We used several different online resources to calculate sample sizes, and we noted that estimates varied with algorithm. Matlab estimates tended to be smaller than with ClinCalc, G-Power or Biomath, and the latter three resources typically provided similar estimates. Our estimates are based upon data from single-sex (female) groups, and estimated group sizes were less than 9 to detect normalisation of microgliosis and astrocytosis and to detect retarded amyloid deposition in these mice. Group sizes required to detect treatment effects in behavioural outcomes equivalent to normalisation to WT levels were up to 22 for younger mice, where impairments were mild, but were smaller for more robust, later behavioural deficits (N ≤ 9). However, sample sizes for preclinical trials should also take into account attrition that may occur during aging and that an unknown agent’s efficacy may be smaller than an effect equivalent to normalisation to WT levels or indeed, a 30% improvement.

Our data replicate a growing literature suggesting 1) that robust cognitive deficits do not develop until 5xFAD TG mice are approximately 1 year of age and 2) that motor hyperactivity and failure to gain weight is a consistent finding in these mice. This pattern of disease progression provides a long window for analysis prior to robust symptomatic disease because these mice show an abnormal plaque load, astrocytosis and microgliosis from approximately 1.5m of age [26]. Patients with autosomal dominant AD show an age of onset from 35–60 years depending upon the causative mutation [94]; thus, although mice typically live for up to 2 years or more in the laboratory, this age of onset of robust deficits (approximately 1 year) in TG 5xFAD mice that carry mutations in APP and PSEN1 is relatively consistent with patients. Considering data from humans, which suggests that more than 50% of disease progression is independent of amyloid [94], these mice remain a representative model for the study of this devastating disease.

## Supporting information

S1 Table Coat colour, used as a surrogate for albinism or pink-eye dilution, has no major impact upon 5xFAD TG performance in cognitive- or activity-based behavioural outcomes. (DOCX)

S1 File Contains data for this paper. (XLSX)
