# Supplementary material for: Preclinical trials in Alzheimer’s disease: Sample size and effect size for behavioural and neuropathological outcomes in 5xFAD mice
Source: PLoS One. 2023 Apr 10;18(4):e0281003. doi: 10.1371/journal.pone.0281003 (PMC10085059; doi:10.1371/journal.pone.0281003)
Supplement: S1 Table — (DOCX) [file pone.0281003.s001.docx]

**S1 Table Coat colour, used as a surrogate for albinism or pink-eye dilution, has no major impact upon 5xFAD TG performance in cognitive- or activity-based behavioural outcomes.**

| **Outcome measure** | **Effect of coat colour?** | **ANOVA results** |
| --- | --- | --- |
| **Young cohort** | | |
| Open field  Total distance moved | No | F (1, 22) = 0.8; p = 0.4  Two-way ANOVA (factors = genotype + coat colour) |
| Open field  Velocity | Yes  Effect observed in WT mice only^a^ | F (1, 22) = 5.9; p = 0.02  Two-way ANOVA (factors = genotype + coat colour) |
| Y maze  Alternations | No | F (1, 26) = 0.2; p = 0.6  Two-way ANOVA (factors = genotype + coat colour) |
| Y maze  WM errors | No | F (1, 26) = 0.2; p = 0.7  Two-way ANOVA (factors = genotype + coat colour) |
| Y maze  Entries | No | F (1, 26) = 0.2; p = 0.6  Two-way ANOVA (factors = genotype + coat colour) |
| Novel object recognition  Preference score | No | F (1, 23) = 0.0005; p = 1.0  Two-way ANOVA (factors = genotype + coat colour) |
| Novel object recognition  Difference score | No | F (1, 23) = 0.06; p = 0.8 (DS)  Two-way ANOVA (factors = genotype + coat colour) |
| Novel object recognition  Discrimination index | No | F (1, 23) = 0.0005; p = 1.0  Two-way ANOVA (factors = genotype + coat colour) |
| Morris water maze  Cued task (Gallagher’s proximity) | No | WT: F (1, 11) = 2.4; p = 0.2  TG: F (1, 14) = 4.2; p = 0.06 Two-way ANOVA (factors = coat colour + training day) |
| Morris water maze  Spatial learning (Gallagher’s proximity) | No | WT: F (1, 11) = 0.3; p = 0.6  TG: F (1, 14) = 0.6; p = 0.5  Two-way ANOVA (factors = coat colour + training day) |
| Morris water maze  Spatial learning: Probe trial (total trial) (Gallagher’s proximity) | No | F (1, 25) = 0.1; p = 0.8  Two-way ANOVA (factors = coat colour + genotype) |
| Morris water maze  Reversal learning (Gallagher’s proximity) | No | WT: F (1, 11) = 0.2; p = 0.7  TG: F (1, 14) = 1.3; p = 0.3  Two-way ANOVA (factors = coat colour + training day) |
| Morris water maze  Reversal learning: Probe trial (total trial) (Gallagher’s proximity) | No | F (1, 25) = 0.2; p = 0.6  Two-way ANOVA (factors = coat colour + genotype) |
|  |  |  |
| **Aged cohort** | | |
| Open field  Total distance moved | No | F (1, 25) = 1.229 ; p = 0.3  Two-way ANOVA (factors = genotype + coat colour) |
| Open field  Velocity | No | F (1, 25) = 0.99; p = 0.3  Two-way ANOVA (factors = genotype + coat colour) |
| Y maze  Alternations | No | F (1, 25) = 1.9; p = 0.2  Two-way ANOVA (factors = genotype + coat colour) |
| Y maze  WM errors | No | F (1, 25) = 1.8; p = 0.2  Two-way ANOVA (factors = genotype + coat colour) |
| Y maze  Entries | Yes  Effect observed in TG mice only^b^ | F (1, 25) = 8.1; p < 0.01  Two-way ANOVA (factors = genotype + coat colour) |
|  |  |  |
| **Both cohorts combined** | | |
| Body weight | No | WT: F (1, 29) = 0.7; p = 0.4  TG: F (1, 26) = 2.6; p = 0.1  Two-way ANOVA (factors = coat colour + age) |

^a^Šídák's multiple comparisons test

^b^Šídák's multiple comparisons test
